# Substrate-dependent competition and cooperation relationships between *Geobacter* and *Dehalococcoides* for their organohalide respiration

**DOI:** 10.1038/s43705-021-00025-z

**Published:** 2021-06-09

**Authors:** Yongyi Liang, Qihong Lu, Zhiwei Liang, Xiaokun Liu, Wenwen Fang, Dawei Liang, Jialiang Kuang, Rongliang Qiu, Zhili He, Shanquan Wang

**Affiliations:** 1 Environmental Microbiomics Research Center, School of Environmental Science and Engineering, Guangdong Provincial Key Laboratory of Environmental Pollution Control and Remediation Technology, Southern Marine Science and Engineering Guangdong Laboratory (Zhuhai), Sun Yat-Sen University, Guangzhou, China; 2 Beijing Key Laboratory of Bio-inspired Energy Materials and Devices, School of Space & Environment, Beihang University, Beijing, China; 3 Institute for Environmental Genomics and Department of Microbiology and Plant Biology, University of Oklahoma, Norman, OK USA; 4 Guangdong Laboratory for Lingnan Modern Agriculture, College of Natural Resources and Environment, South China Agricultural University, Guangzhou, China

**Keywords:** Environmental microbiology, Microbial ecology

## Abstract

Obligate and non-obligate organohalide-respiring bacteria (OHRB) play central roles in the geochemical cycling and environmental bioremediation of organohalides. Their coexistence and interactions may provide functional redundancy and community stability to assure organohalide respiration efficiency but, at the same time, complicate isolation and characterization of specific OHRB. Here, we employed a growth rate/yield tradeoff strategy to enrich and isolate a rare non-obligate tetrachloroethene (PCE)-respiring *Geobacter* from a *Dehalococcoides*-predominant microcosm, providing experimental evidence for the rate/yield tradeoff theory in population selection. Surprisingly, further physiological and genomic characterizations, together with co-culture experiments, revealed three unique interactions (i.e., free competition, conditional competition and syntrophic cooperation) between *Geobacter* and *Dehalococcoides* for their respiration of PCE and polychlorinated biphenyls (PCBs), depending on both the feeding electron donors (acetate/H_2_ vs. propionate) and electron acceptors (PCE vs. PCBs). This study provides the first insight into substrate-dependent interactions between obligate and non-obligate OHRB, as well as a new strategy to isolate fastidious microorganisms, for better understanding of the geochemical cycling and bioremediation of organohalides.

## Introduction

Organohalides are organic compounds in which carbons are linked to halogens by covalent bonds. In natural pristine environments, organohalides as an indispensable portion of the halogen biogeochemical cycle are formed by living organisms (e.g., bacteria, fungi, and plants), biomass burning, volcanic activities and other geothermal processes.^1^ Thus far, more than 5000 biogenic or geogenic organohalides have been chemically identified and characterized, including around 2300 organochlorines and 2050 organobromines.^2,3^ Nonetheless, in the past few decades, both diversity and abundance of organohalides in natural environments have been changed due to the anthropogenic organohalides, which have been massively produced for industrial and agricultural purposes, including PCE and PCBs. Particularly, the improper handling and disposal of anthropogenic organohalides have resulted in their worldwide environmental occurrence, bioaccumulation and biomagnification via food webs, and consequent side effects on the public health and ecosystem functions.^4–6^

**Fig. 1:**
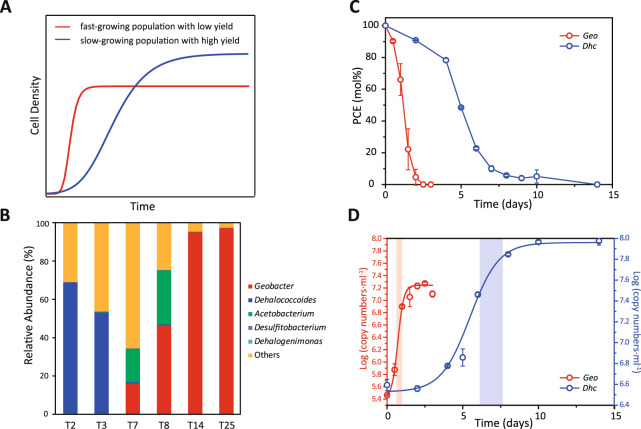
Selective enrichment of *Geobacter lovleyi* LYY based on the cell growth rate/yield tradeoff of *Geobacter* and *Dehalococcoides*. **A** The rate/yield tradeoff-based growth curves of fast-growing population with low cell density (red line) and slow-growing population with high cell density (blue line). **B** Changes in microbial community composition when shifting the selective enrichment from growth-yield selection to growth-rate selection by shortening culture incubation time. T2 and T25 represent 2nd and 25th culture transfers, respectively. See Fig. S2 for detailed community composition data. Dechlorination of PCE (**C**) and corresponding cell growth (**D**) of pure cultures *Geobacter lovleyi* LYY (Geo, red line) and *Dehalococcoides mccartyi* CG1 (Dhc, blue line) to show their growth rate/yield tradeoff. Error bars represent SDs of triplicate cultures. Optimum transfer time of strains LYY and CG1 are shaded in red and blue colors, respectively.

In the long-term evolution by natural selection, phylogenetically diverse bacteria are able to obtain energy for cell growth through organohalide respiration, in which electrons are derived and transported from organics or hydrogen to organohalides via cytoplasmic-membrane associated electron transport chains.^7,8^ These organohalide-respiring bacteria (OHRB) can be further classified as obligate and non-obligate OHRB.^9^ In contrast to the flexible respiratory electron transport chains of non-obligate OHRB (e.g., *Geobacter*, *Desulfitobacterium*, *Desulfuromonas*, and *Sulfurospirillum*), obligate OHRB (e.g., *Dehalococcoides* and *Dehalogenimonas*) are restricted to reductive dehalogenation as a terminal electron-accepting process in their respiration.^8,10^ In addition, the obligate and non-obligate OHRB may coexist in a community where they potentially compete for organohalides as respiratory electron acceptors.^2,9,11,12^ Since most OHRB may not be able to grow on a solid medium in laboratory, their isolation requires the enrichment as a predominant population prior to their subsequent serial dilution to extinctions in a liquid medium.^13^ The coexistence of obligate and non-obligate OHRB, particularly their complicated interactions, may enormously increase the difficulty in their isolation. Nonetheless, in-depth insights into interactions between, as well as characteristics of, the obligate and non-obligate OHRB may facilitate their isolation and subsequent characterization and application. Taking the cell growth rate and yield of OHRB as an example, many non-obligate OHRB (e.g., *Geobacter* and *Desulfitobacterium*) grow faster but with lower cell yield compared to obligate OHRB (e.g., *Dehalococcoides*) (Table S1). According to the rate/yield tradeoff underlying the selection of specific populations,^14^ long cultivation time may favor the growth of slow-growing but high yield populations, e.g., *Dehalococcoides*. By contrast, non-obligate OHRB with high growth rate and low cell yield are beneficial at a low population density with high food availability at the early inoculation stage (Fig. 1A). Consequently, the rate/yield tradeoff theory may be employed to isolate specific populations from complicated microbial communities, which awaits experimental evidences.

In dechlorinating microbial communities, obligate OHRB like *Dehalococcoides* and *Dehalogenimonas* need to form syntrophic metabolic networks with other microorganisms,^15–18^ specifically the non-obligate OHRB and non-dehalogenating microorganisms (non-OHRB). The latter provide electron donor, carbon source and other essential nutrients or growth cofactors to support organohalide respiration of the obligate OHRB. For example, both cultivation and metagenomics evidence suggest that *Desulfovibiro* and *Methanosarcina* can provide and balance the availability of acetate and hydrogen as carbon source and electron donor, respectively, for *Dehalococcoides.*^17,18^ Consequently, it is rational to hypothesize that substrates (e.g., electron donors and acceptors) may shape organohalide-respiring community assemblies by regulating both the microbial community composition and function, in particular, the interactions among obligate OHRB, non-obligate OHRB and non-OHRB. Although interactions between obligate and non-obligate OHRB have profound impacts on the organohalide geochemical cycling, information on their interactions remain elusive, in contrast to extensive investigations on the syntrophy between OHRB and non-OHRB.^15–18^ In addition, the missing information may result in unpredictable in situ dehalogenation activity of OHRB and consequently failed bioremediation of organohalide pollution.^16^

In this study, based on the competition between obligate and non-obligate OHRB, we employed the growth rate/yield tradeoff theory to isolate a PCE-dechlorinating *Geobacter* from a *Dehalococcoides*-predominant PCB/PCE-dechlorinating microcosm. Further physiological, genomic characterizations and co-culture experiments suggest three unique substrate-dependent interactions between *Geobacter* and *Dehalococcoides* for the organohalide respiration:^1^ free competition for both the electron donor and acceptor in medium amended with acetate/H_2_ and PCE;^2^ conditional competition for PCE as an electron acceptor in medium fed with propionate and PCE (in this scenario, *Geobacter* and *Dehalococcoides* compete for PCE as the electron acceptor and, at the same time, *Geobacter* provides acetate and H_2_ for *Dehalococcoides*);^3^ syntrophic cooperation for stepwise electron transfer from the donor to the acceptor in medium fed with propionate and PCBs. This study opens a new avenue for isolation of fastidious microorganisms, and provides unprecedented insights into the previously underestimated interactions between obligate and non-obligate OHRB in the halogen biogeochemical cycle.

## Materials and methods

### Culture medium and enrichment

A sediment-free PCE- and PCB-dechlorinating culture LY established with black-odorous urban river sediment was employed to enrich a low-abundance PCE-dechlorinating *Geobacter*, in which *Dehalococcoides* was present as a predominant OHRB.^19,20^ Cultures were transferred in a defined anaerobic mineral salts medium with a headspace of N_2_/CO_2_ (80:20, v/v).^21,22^ The medium was fed with lactate (10 mM) or acetate (10 mM) as a carbon source, hydrogen (5 × 10^4^ Pa) as an electron donor and PCE (1 mM) as an electron acceptor. To control redox potential in the medium, L-cysteine (0.024 g/L) and Na_2_S (0.048 g/L) were amended as reducing agents, and resazurin (0.005 g/L) was added as a redox indicator. The PCE- and PCB-dechlorinating culture LY was first transferred (1%, v/v) in the medium amended with lactate and PCE. After five successive transfers, the growth rate/yield tradeoff strategy was employed to enrich *Geobacter* by shortening incubation time from 8 days to 3 days, and the carbon source and electron donor was changed from lactate to acetate and hydrogen. After 13th culture transfer, ampicillin (50 mg/L) was spiked into the culture medium to enrich ampicillin-tolerant *Geobacter* and to control cell growth of nonresistant microorganisms. Unless stated otherwise, the cultures were incubated at 30 °C in the dark without shaking.

### Isolation and characterization of *Geobacter lovleyi* LYY

The *Geobacter*-enriched culture was subjected to serial dilutions in 20 ml vials filled with 10 ml of medium amended with acetate (10 mM), hydrogen (5 × 10^4^ Pa), PCE (1 mM) and ampicillin (50 mg/L). PCE dechlorination activities were repeatedly detected in 10^-7^/10^-8^ dilution vials, which were employed to inoculate subsequent serial dilutions. After 17 times of serial dilutions, a culture dechlorinating PCE to *cis*-DCE was obtained, of which culture purity was confirmed via scanning electron microscope (SEM) and genome sequencing analyses. The isolated strain LYY was deposited to Guangdong Microbial Culture Collection Center (GDMCC) with an accession number of GDMCC 1.1621. In the pure culture, varied carbon source, electron donors and electron acceptors were tested to verify whether they supported the growth of strain LYY, i.e., 10 mM formate, 10 mM acetate, 10 mM propionate, 10 mM butyrate, 10 mM lactate, 10 mM citrate, 10 mM pyruvate, or 10 mM glucose as a carbon source and/or electron donor; 0.25 mM chloroethenes, 0.1 mM Fe(III), 0.1 mM nitrate, 0.1 mM nitrite, or 0.1 mM sulfate as an electron acceptor (Table S2). Inhibitive effects of PCE and ampicillin were tested in pure cultures amended with gradient concentrations of PCE (0.25–5 mM) or ampicillin (0–1000 mg/L). To test interactions between obligate and non-obligate OHRB, three sets of experiments were established with pure cultures of *Geobacter lovleyi* LYY and *Dehalococcoides mccartyi* CG1, and a co-culture of *Geobacter lovleyi* LYY and *Dehalococcoides mccartyi* CG1 fed with different substrates:^1^ 10 mM acetate, 5 × 10^4^ Pa H_2_ and 1 mM PCE;^2^ 10 mM propionate and 1 mM PCE;^3^ 10 mM propionate and 2.5 μM PCB180. All experiments were setup in triplicates and incubated in the dark at 30 °C without shaking.

### Cell morphology

Cell morphology was observed with SEM (Sigma 500, Zeiss, Germany). Samples for SEM observations were collected on day 10 and day 60 from PCE- and PCB180-dechlorinating Dhc-Geo cocultures, respectively, and on day 3 from PCE-dechlorinating *Geobacter* pure culture. Sample fixation and dehydration for SEM observation were performed as described previously.^23^ Briefly, samples were first immersed in glutaraldehyde (2.5%, v/v) overnight. After rinsing in phosphate-buffered saline (0.1 M, pH 7.3) for three times (10 min each), the samples were dehydrated with gradient concentrations of ethanol (30, 50, 70, 85, 95, and 100%, v/v) to prevent dehydration-induced shrinkage. After that, pure tertiary butyl alcohol (TBA) was applied to achieve substitution of ethanol in microbial cells. Finally, 0.2 μm polyether sulfone (PES) membrane-filtered samples (Supor 200 Membrane disc filter, Pall) were observed at the acceleration voltage of 3 kV.

### Analytical techniques

Headspace samples of chloroethenes were analyzed on a gas chromatograph (Agilent 7890B, Wilmington, DE, USA) equipped with a flame ionization detector and a Gas-Pro capillary column (30 m × 0.32 mm; Agilent J&W Scientific, Folsom, CA, USA) as described.^13,20,24^ PCB samples were extracted with isooctane and quantified by the same model GC equipped with an electron capture detector and an HP-5 capillary column (30 m × 0.32 mm × 0.25 µm film thickness; Agilent J&W Scientific, Folsom, CA, USA) as described previously.^13,20,24^

### DNA extraction, PCR amplification, qPCR, and microbial community analyses

Cells for genomic DNA extraction were harvested by centrifugation (15 min, 10,000 × *g*, 4 °C) when cells reached the exponential growth phase. Genomic DNA for PCR/qPCR and genome sequencing analyses were extracted from 1 mL and 500 mL cultures, respectively, using the FastDNA Spin Kit for soil (MP Biomedicals, Carlsbad, CA, USA). The 16S rRNA gene amplicon sequencing-based microbial community analyses were performed with Quantitative Insights Into Microbial Ecology (QIIME, v1.9.1) as described.^24^ Briefly, sequences with a distance-based similarity of ≥97% were grouped into operational taxonomic units (OTUs), and OTU taxonomy was assigned with RDP Classifier with a confidence cutoff of 80% (SLIVA V132 database). The phylogenetic trees were constructed with the neighbor-joining method (MEGA 7).^25^ The qPCR (CFX96 Touch System; Bio-Rad, CA, USA) enumeration of *Geobacter* and *Dehalococcoides* cells was performed with QuantiTect SYBR Green PCR kit as described.^13,26,27^

### Genome sequencing, assembly, and annotation

DNA sequencing library construction and subsequent Illumina HiSeq sequencing services were provided by BGI (Shenzhen, China). The genome sequencing raw data were filtered to remove low quality bases/reads using Sickle,^28^ with parameters set to “−q = 20 and −l = 100”. de novo contig assembly was performed with SPAdes (version 3.12.0),^29^ and different k-mer sizes (i.e., 77, 99, and 121) were tried. High quality reads were first mapped with Bowtie2 (version 2.3.4.3).^30^ Then, Samtools (version 1.9)^31^ was used for converting the bowtie output to a sorted and indexed bam file. The contigs of assembly was extended and polished with Geneious (version 10.22) as described,^32^ which generated 14 contigs. Completeness and contamination of generated draft genome was evaluated using CheckM.^33^ Protein coding sequences (CDS) were determined using prokka (version 1.12)^34^ with the “--quiet” option. To reconstruct the metabolic pathways, CDS were annotated at the KEGG automatic annotation server (KAAS) as described.^35^ The retrieved metabolic potential of strain LYY was further manually curated. The genomic SNP-based analyses were performed as described.^13^ Briefly, strain LYY’s genome sequencing reads were first mapped to the reference genome of *Geobacter lovleyi* SZ. Then, LoFreq was employed to call genome-wide SNPs with default parameters and assign frequencies to each SNP.

## Results

### Competitive growth of *Geobacter* and *Dehalococcoides*

A sediment-free PCB/PCE-dechlorinating culture LY was established with polluted urban river sediment, which dechlorinated PCE to ethene via trichloroethene (TCE), dichloroethenes (DCEs) and vinyl chloride in two stages (Fig. S1A): (i) PCE-to-DCEs dechlorination without notable formation of TCE in the first 5 days; and (ii) DCEs-to-ethene dechlorination in another 20 days. The DCEs-to-ethene dechlorination were mediated by OHRB belonging to the ethene-generating class Dehalococcoidia,^36^ which was confirmed to be *Dehalococcoides* through microbial community composition analysis (Fig. S2). Since PCE dechlorination by *Dehalococcoides* was generally accompanied with the *trans*-DCE generation, the rapid and predominant PCE-to-*cis*-DCE dechlorination in culture LY suggested the potential involvement of non-obligate OHRB, probably together with PCE-to-DCEs dechlorinating *Dehalococcoides*.

The microbial community composition analysis showed the predominance of *Dehalococcoides* (68.87% in relative abundance), as well as *Geobacter* as a minor non-obligate OHRB (0.35% in relative abundance), in the PCE-dechlorinating culture LY (Fig. 1B, Fig. S2). Interestingly, experimental data from both this study (Fig. 1C and D) and previous studies (Table S1) showed that *Dehalococcoides* generally grew slowly with high cell yield, compared to fast-growing non-obligate OHRB (e.g., *Geobacter*) with low cell yield on PCE, being in line with the rate/yield tradeoff theory^14^ underlying the selection of specific populations. According to the hypothesis of the growth rate/yield tradeoff between *Geobacter* and *Dehalococcoides* (Fig. 1A), the *Geobacter* with high growth rate should benefit at low population densities and high substrate (i.e., PCE) availability. By contrast, at high population densities, PCE became scarce and its economic use by *Dehalococcoides* was favored. Consequently, with long culture incubation time,  the enrichment process favored the growth of *Dehalococcoides* as observed in the culture LY (Fig. 1D). In addition, under the condition with long culture incubation time, *Geobacter* needed to spend longer periods under starvation conditions, which by itself potentially even further favored the selection of slow-growing *Dehalococcoides*. When shortening culture incubation time from around 8 days to 3 days (Fig. 1D), the growth rate/yield tradeoff-based selective enrichment changed the dominant OHRB from *Dehalococcoides* to *Geobacter* (Fig. 1B). For example, with continuous employment of the short transfer time, *Dehalococcoides* phased out from the culture LY and *Geobacter* increasingly became a dominant population (97.4% in relative abundance) after 25 times of culture transfers (Fig. 1B, Fig. S2).

### Isolation and characterization of *Geobacter lovleyi* LYY

The enrichment culture was further subjected to serial dilution-to-extinctions in the defined medium amended with ampicillin, acetate as a carbon source, H_2_ as an electron donor and PCE as an electron acceptor. This resulted in successful isolation of a *Geobacter lovleyi* LYY, of which the purity was confirmed by scanning electron microscope (SEM) (Fig. S3A) and SNP-based genome sequencing analyses (Fig. S3B). The SEM revealed uniform rod-shaped morphology of strain LYY with a size of 1–3 µm in length and 0.2–0.5 µm in diameter. Strain LYY shared 99% 16S rRNA gene sequence identity (over 1450 bp) with *G. lovleyi* SZ (Fig. S4). Similar to strain SZ, strain LYY dechlorinated PCE to *cis*-DCE via TCE in 4 days (Fig. S1B) and respired on ferric iron and nitrate as alternative electron acceptors (Table S2). Notably, strain LYY used acetate as both an electron donor and carbon source, which was consequently transferred in the medium without hydrogen amendment. Moreover, strain LYY grew in the medium amended with high concentrations of PCE (0.25–2 mM; Fig. S5A) and ampicillin (0–250 mg/L; Fig. S5B), and their dechlorination lag phases were remarkably prolonged when further increasing concentrations of PCE from 2 mM to 3 mM and ampicillin from 250 to 500 mg/L. The highest PCE concentration to support strain LYY’s growth was close to chloroethene concentrations at contaminated sites (3–7 mM),^37,38^ and much higher than the concentrations in laboratory experiments (0.01–0.6 mM).^13,21,36,39,40^ These properties, together with aforementioned high cell growth rate but low yield, might confer *Geobacter* with advantages in competition with *Dehalococcoides* for PCE dechlorination as observed at PCE nonaqueous phase liquid source zones at contaminated sites.^41^

**Fig. 2:**
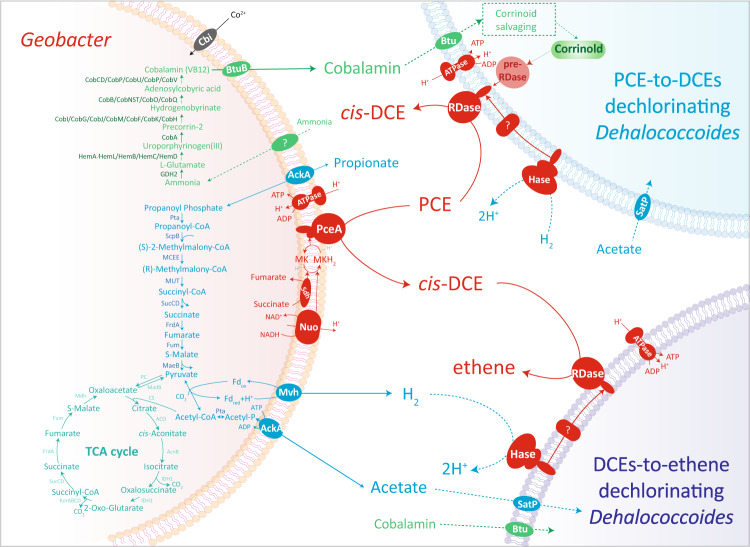
Predicted metabolic networks in a PCE-dechlorinating microcosm containing *Geobacter* and *Dehalococcoides*. The predicted metabolic networks were proposed based on genomes of *Geobacter lovleyi* LYY, *Dehalococcoides mccartyi* CG1 and VS, including organohalide respiration (red), propionate-to-acetate/hydrogen metabolism (blue), TCA cycle (cyan) and cobalamin synthesis and transport (green).

The assembled draft genome of strain LYY was 3,670,069 bp long (Fig. S6A), with one 81,281 bp plasmid (Fig. S7) and a GC content of 54.9% (Fig. S6A). CheckM results showed that the completeness of strain LYY’s draft genome was estimated to be 99.07%. The chromosome contained 3521 protein-coding genes, a number similar to those of *G. lovleyi* SZ, *Geobacter thiogenes* K1, and *Geobacter sulfurreducens* PCA (Fig. S6A). In contrast to two reductive dehalogenases (RDase) homolog (*rdh*) genes in the genome of *G. lovleyi* SZ, the draft genome of strain LYY had one single full-length *rdhA* gene, sharing a 99% amino acid sequence similarity with PceA of *G. lovleyi* SZ (Fig. S6B). Consequently, the only *rdhA* gene of strain LYY was the key functional gene encoding PCE RDase for PCE dechlorination by strain LYY (Fig. S6C). The genome encoded enzymes for propionate and acetate metabolism, as well as for multidrug efflux, which corroborated the strain’s flexible carbon utilization and high tolerance to ampicillin (Fig. 2, Fig. S8, Table S3). Interestingly, the genome-encoded pathway from propionate to acetate and hydrogen suggested that strain LYY might support organohalide respiration of *Dehalococcoides* with carbon source and electron donor in environments without already-available acetate and hydrogen (Fig. 2). In addition, based on the gene-encoding metabolic potentials, strain LYY might compete with PCE-to-DCEs dechlorinating *Dehalococcoides* for PCE but support organohalide respiration of DCEs-to-ethene dechlorinating *Dehalococcoides* by providing *cis*-DCE (Fig. 2). Moreover, the cobalamin biosynthesis and transport genes in the plasmid (Fig. S7, Table S4) implied that *Geobacter* potentially provided cobalamin source for the microorganisms (e.g., *Dehalococcoides*) without de novo cobalamin-synthesizing capability (Fig. 2).

### Substrate-dependent competition and collaboration between *Geobacter* and *Dehalococcoides*

**Fig. 3:**
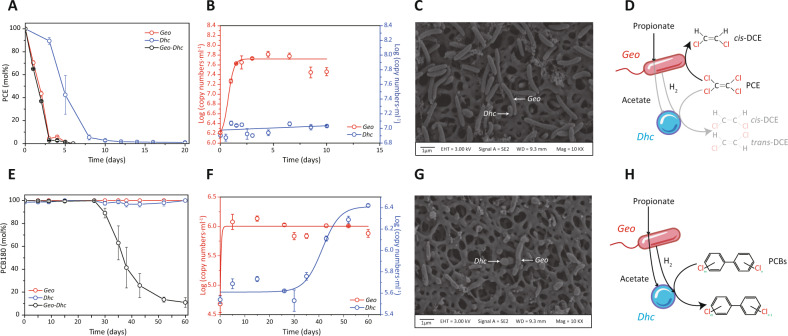
Substrate-dependent competition and cooperation interactions between *Geobacter* and *Dehalococcoides*. **A** PCE dechlorination in *G. lovleyi* LYY (Geo, red line), *D. mccartyi* CG1 (Dhc, blue line) pure cultures and Geo-Dhc co-culture (black line). Cell growth (**B**) and representative SEM images (**C**) of *G. lovleyi* LYY and *D. mccartyi* CG1 in the PCE-dechlorinating co-culture. **D** A proposed competition relationship between *Geobacter* and *Dehalococcoides* in their PCE- and propionate-fed co-culture. **E** PCB dechlorination in *G. lovleyi* LYY (Geo, red line), *D. mccartyi* CG1 (Dhc, blue line) pure cultures and Geo-Dhc co-culture (black line). Cell growth (**F**) and representative SEM images (**G**) of *G. lovleyi* LYY and *D. mccartyi* CG1 in the PCB-dechlorinating co-culture. **H** A proposed cooperation relationship between *Geobacter* and *Dehalococcoides* in their PCB- and propionate-fed co-culture. Error bars represent SDs of triplicate cultures.

Aforementioned competitive enrichment and genome sequencing of strain LYY implied that *Geobacter* and *Dehalococcoides* might form not only competition but cooperation relationships between them. To gain insights into detailed interactions, two batches of experiments were conducted with a co-culture constituted of two isolates, i.e., PCE-dechlorinating *G. lovleyi* LYY (Geo) and PCE/PCB-dechlorinating *D. mccartyi* CG1 (Dhc): (i) PCE-dechlorinating co-culture fed with propionate, together with PCE-dechlorinating pure cultures LYY and CG1 fed with propionate and acetate/hydrogen, respectively, as controls (Fig. 3A–D); (ii) PCB180-dechlorinating co-culture fed with propionate, as well as pure cultures LYY and CG1 amended with propionate and PCB180 as controls (Fig. 3E–H). In the first batch of experiments, similar with PCE dechlorination in pure culture LYY, PCE in the co-culture was dechlorinated to *cis*-DCE in three days (Fig. 3A, Fig. S9), suggesting the predominance of *Geobacter* in the PCE dechlorination. Quantification of bacterial cells of strains LYY and CG1 in the PCE-dechlorinating co-culture showed the coupled growth of *Geobacter* with PCE dechlorination (Fig. 3B), i.e., 9.1 × 10^7^ and 1.2 × 10^6^ 16 S rRNA gene copies per ml of strains LYY and CG1, respectively. SEM analysis corroborated the predominance of *Geobacter* in the PCE-dechlorinating co-culture (Fig. 3C). All of those experimental evidences worked together to generate a scenario for competitive PCE dechlorination in the propionate-fed co-culture (Fig. 3D): *Geobacter* used propionate as both carbon source and electron donor to dechlorinate PCE and, at the same time, supported organohalide respiration of *Dehalococcoides* with acetate and hydrogen. In the competitive PCE dechlorination, *Geobacter* harvested energy from both PCE respiration (major portion; Fig. 3B) and propionate-to-acetate/hydrogen metabolism (minor portion; Fig. 3F). Consequently, both the low dechlorination rate and derivation of acetate/hydrogen from *Geobacter* resulted in *Dehalococcoides*’ loss of competitive edge in the co-culture.

In contrast to the PCE dechlorination within 5 days (Fig. 3A), dechlorination of PCB180 (2345-245-CB) in the co-culture took around 60 days (Fig. 3E). No PCB dechlorination activity was observed in the *Dehalococcoides* and *Geobacter* pure cultures amended with propionate and PCB180 (Fig. 3E). In the co-culture, only *Dehalococcoides* coupled growth with reductive dechlorination of PCB180 (Fig. 3F). By contrast, *Geobacter* increased from 5.0 × 10^4^ to 1.3 × 10^7^ 16S rRNA gene copies per ml in the first 5 days, and potentially derived the anabolic energy from propionate-to-acetate/hydrogen catabolism, of which the cell number was much lower than its abundance in PCE-dechlorination experiments (Fig. 3B). In line with qPCR quantitation data, SEM showed the slightly more but comparable cells of *Dehalococcoides* relative to *Geobacter* (Fig. 3G). Consequently, in the PCB-dechlorinating co-culture, *Dehalococcoides* and *Geobacter* formed a syntrophic cooperation relationship (Fig. 3H): *Geobacter* first derived energy from the conversion of propionate into acetate and hydrogen, and then *Dehalococcoides* employed the already-available carbon source and electron donor to dechlorinate PCBs. Such continuous consumption of acetate and hydrogen by *Dehalococcoides* further facilitated the syntrophic acetogenesis of *Geobacter*.

## Discussion

**Fig. 4:**
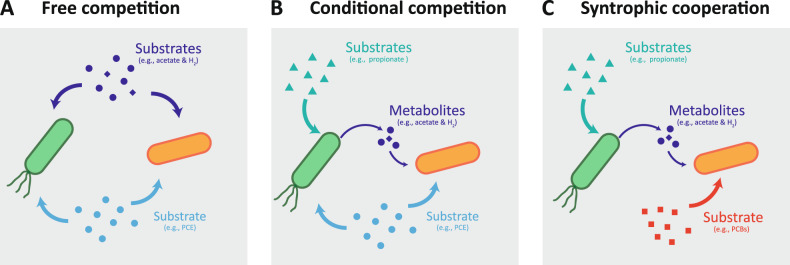
A conceptual model for interactions between obligate and non-obligate OHRB. **A** Free competition, the obligate and non-obligate OHRB compete with each other for the carbon source, electron donor and acceptor. **B** Conditional competition, the non-obligate OHRB provide carbon source and electron donor, as well as cobalamin if necessary, for the obligate OHRB, but both populations need to compete for the same electron acceptor. **C** syntrophic cooperation, the obligate OHRB need to derive carbon source and electron donor from non-obligate OHRB for organohalide respiration, and consequently facilitate the syntrophic acetogenesis of the non-obligate OHRB by removing metabolites in the medium in which the organohalide and carbon source can be exclusively used by the obligate and non-obligate OHRB, respectively.

The relationships between OHRB have been generally based on competition.^9,12,42^ This study, for the first time, reports a unique substrate-dependent competition- and cooperation-relationship between *Geobacter* and *Dehalococcoides* for their organohalide respiration (Fig. 4): (i) free competition, *Geobacter* and *Dehalococcoides* competitively grow in the medium amended with acetate, hydrogen and PCE, and their competition edge depends on the rate/yield tradeoff, similar to the case of enriching *G. lovleyi* LYY from the *Dehalococcoides*-predominant microcosm (Fig. 4A); (ii) conditional competition, *Geobacter* and *Dehalococcoides* competitively grow in the medium amended with propionate and PCE, and *Dehalococcoides* need to derive acetate and hydrogen from *Geobacter* and, at the same time, both *Geobacter* and *Dehalococcoides* compete for PCE as an electron acceptor (Fig. 4B); and (iii) syntrophic cooperation, *Geobacter* and *Dehalococcoides* synergistically grow in the medium amended with propionate and PCBs, and *Dehalococcoides* need to derive acetate and hydrogen from *Geobacter* for PCB dechlorination and, at the same time, remove metabolic products from the medium to increase energy gain of *Geobacter* from syntrophic acetogenesis (Fig. 4C, Table S5). The thermodynamically syntrophic propionate catabolism of *Geobacter* and *Dehalococcoides* is similar with the syntrophic aromatic compound degradation in methanogenic reactors. Therefore, growth substrates can shape the organohalide-respiring community assembly through mediating the interactions among different groups of OHRB. In addition, detailed information on the interactions can make dechlorination activities of OHRB predictable and guide microbiome engineering for remediation of organohalide pollution. Notably, the substrate-dependent interactions between *Geobacter* and *Dehalococcoides* are proposed based on the cultivation and genomic data, which may be further corroborated using other meta-omics analyses.

*Geobacter* generally mediate organic and metal reductions with a wide range of redox potential and are ubiquitous in soil, sediments and subsurface environments (Table S6) where *Dehalococcoides* may inhabit and respire on organohalides.^43,44^ Consequently, the competition and cooperation relationships between *Geobacter* and *Dehalococcoides* can be widely present in natural environments, which has been underappreciated in previous studies. In addition, there are many bacterial lineages sharing similar metabolic traits and ecological niches with non-obligate organohalide-respiring *Geobacter* and obligate organohalide-respiring *Dehalococcoides*. Similar to *Geobacter*, non-obligate OHRB of Firmicutes (e.g., *Desulfitobacterium*) and Proteobacteria (e.g., *Sulfurospirillum*, *Desulfuromonas*, *Desulfoluna,* and *Anaeromyxobacter*) have very flexible respiratory electron transport chains enabling them to grow under different environmental conditions.^8,40,45–48^ For example, the PCE-to-*cis*-DCE dechlorinating *Sulfurospirillum multivorans* (DSM 12446^T^) can derive electrons from hydrogen, formate, pyruvate and NADH by employing H_2_ dehydrogenase (or pyruvate oxidase, Pox) or NADH dehydrogenase (Nuo) to support organohalide respiration.^45,49,50^ The obligate organohalide-respiring *Dehalogenimonas* of Dehalococcoidia class share similar metabolic traits with *Dehalococcoides* of the same class. Therefore, similar substrate-dependent competition and cooperation relationships can be established between many non-obligate and obligate OHRB in natural environments.

Enrichment and isolation of yet-to-be-cultured microorganisms can be challenging,^51,52^ particularly the enrichment of anammox, comammox, and functional microorganisms removing halogens from halogenated persistent organic pollutants.^13,53^ There are many strategies being devised based on specific properties of targeting microorganisms, e.g., isolation of PCB-dechlorinating *Dehalococcoides* with PCE as an alternative organohalide^13^ and single cell-based bacterial enrichment and characterization using microfluidic chips.^54^ In this study, we employ the theory of tradeoff between growth rate and biomass yield of two competing populations to successfully isolate a rare organohalide-respiring *Geobacter* from a *Dehalococcoides*-predominant microcosm. The rate/yield tradeoff can be linked to metabolic strategies of a specific population under different growth conditions (e.g., switch between respiration and fermentation in yeast), and also provide a rationale for the outcome of natural population selection in a microbial community.^14,55^ In this study, at the beginning, the slow-growing *Dehalococcoides* outcompete *Geobacter* in the prolonged PCE-dechlorinating microcosm, but the fast-growing *Geobacter* wins the competitive edge at the cost of biomass yield when shortening the culture incubation time. Consequently, the strategy based on rate/yield tradeoff employed in this study opens a new avenue for the isolation of yet-to-be-cultured microorganisms. Moreover, since it is extremely challenging to isolate a population from their cooperative partners, it is necessary to elucidate interactions of the targeting population with other microorganisms and to prevent their syntrophic catabolism. Consequently, prerequisites of employing the rate/yield tradeoff theory in isolation of strain LYY include:^1^ to elucidate the interaction of non-obligate OHRB (i.e., *Geobacter*) and obligate OHRB (i.e., *Dehalococcoides*);^2^ to transfer the interaction from cooperation to competition by changing substrate. In addition, this study provides experimental evidence for the rate/yield tradeoff theory in population selection, of which the experimental evidence under controlled conditions remains scarce.^14,56^ Notably, the negative correlation between population growth rate and biomass yield has been frequently observed in microbial communities of varied environmental niches.^14,57,58^ Take the microbial-mediated nitrogen cycling for example, comammox *Nitrospira* has lower growth rates but higher biomass yield compared to other characterized ammonia-oxidizing bacteria and non-marine ammonia-oxidizing archaea.^57,58^ Therefore, the rate/yield tradeoff theory may be employed to enrich and isolate populations like the comammox, which warrants future studies.

## Supplementary information

Supplementary Figures

Supplementary Tables

## Data Availability

Raw Illumina Hiseq sequencing reads for 16S rRNA gene amplicons and strain LYY’s genome were deposited into the NCBI sequence read archive with accession numbers of PJRNA628620 and PRJNA626355, respectively. The 16S rRNA gene amplicon Sanger sequencing data of strain LYY were deposited to the Genbank with an accession number of MK850090.1. Other data that support the findings of this study are available from the corresponding author upon reasonable request.
