# Supplementary material for: Substrate-dependent competition and cooperation relationships between Geobacter and Dehalococcoides for their organohalide respiration
Source: ISME Commun. 2021 Jun 9;1:23. doi: 10.1038/s43705-021-00025-z (PMC9723705; doi:10.1038/s43705-021-00025-z)
Supplement: Supplementary file 1 — Supplementary Figures [file 43705_2021_25_MOESM1_ESM.pdf]

Supplementary Figures for

# Substrate-dependent competition and cooperation relationships between *Geobacter* and *Dehalococcoides* for their organohalide respiration

Yongyi Liang, Qihong Lu, Zhiwei Liang, Xiaokun Liu, Wenwen Fang, Jialiang Kuang, Dawei Liang, Rongliang Qiu, Zhili He and Shanquan Wang*

Corresponding author: Shanquan Wang (wangshanquan@mail.sysu.edu.cn)

The PDF file includes:

Fig. S1. Reductive dechlorination of PCE.
Fig. S2. The microbial community composition of PCE-dechlorinating microcosms in different transfers.
Fig. S3. Conformation of culture purity with SEM and SNP profiling analyses.
Fig. S4. Phylogeny of *Geobacter lovleyi* LYY.
Fig. S5. PCE dechlorination in *Geobacter lovleyi* LYY pure cultures.
Fig. S6. Genomic characterization of *Geobacter lovleyi* LYY.
Fig. S7. A plasmid map of *Geobacter lovleyi* LYY.
Fig. S8. Predicted metabolic capabilities of *Geobacter lovleyi* LYY.
Fig. S9. PCE dechlorination in a *Geobacter-Dehalococcoides* co-culture.

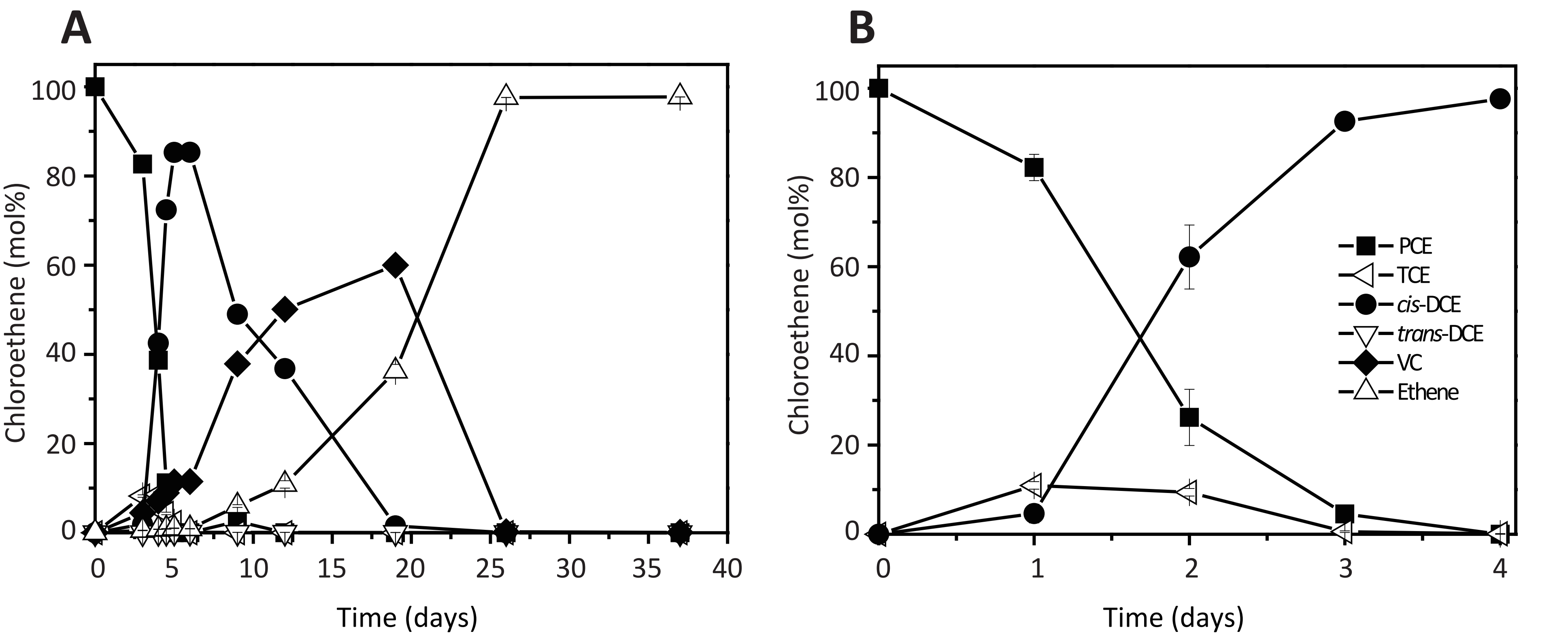


**Fig. S1. Reductive dechlorination of PCE. (A)** PCE dechlorination in microcosm LY (T1). **(B)** PCE dechlorination in *Geobacter lovleyi* LYY pure culture. Error bars represent SDs of triplicate cultures.

| Taxonomy | | Relative abundances (%) of predominant genera | | | | | |
|---|---|---|---|---|---|---|---|
| | | T2 | T3 | T7 | T8 | T14 | T25 |
| Bacteroidetes | *Lentimicrobium* | 1.92 | 14.18 | 2.92 | 0.75 | 0.00 | 0.00 |
| | Blvii28 | 0.40 | 0.02 | 0.09 | 0.23 | 0.00 | 0.00 |
| | Bacteroidales_unculture | 1.92 | 0.13 | 0.46 | 0.13 | 0.01 | 0.01 |
| | Rikenellaceae_unculture | 0.01 | 0.03 | 0.05 | 0.04 | 0.01 | 0.00 |
| Chloroflexi | ***Dehalococcides*** | 68.87 | 52.61 | 0.65 | 0.39 | 0.07 | 0.05 |
| | *Dehalogenimonas* | 0.00 | 0.05 | 0.00 | 0.00 | 0.00 | 0.00 |
| Cloacimonetes | *Ca. Cloacimonas* | 3.51 | 0.15 | 0.03 | 0.03 | 0.00 | 0.00 |
| Firmicutes | *Acetobacterium* | 0.33 | 0.41 | 17.76 | 28.29 | 0.05 | 0.05 |
| | *Clostridium* | 0.25 | 0.25 | 0.48 | 0.35 | 0.21 | 0.18 |
| | *Tyzzerella* | 0.16 | 0.04 | 30.04 | 0.15 | 0.00 | 0.00 |
| | *Soehngenia* | 0.16 | 0.19 | 0.18 | 0.17 | 0.00 | 0.00 |
| | *Acidaminobacter* | 0.17 | 0.10 | 2.11 | 0.06 | 0.02 | 0.01 |
| | *Syntrophomonas* | 0.78 | 0.02 | 0.06 | 0.06 | 0.01 | 0.01 |
| | *Pelotomaculum* | 1.11 | 0.01 | 0.01 | 0.01 | 0.00 | 0.00 |
| | *Youngiibacter* | 0.43 | 0.22 | 0.47 | 0.58 | 0.00 | 0.00 |
| | *Sedimentibacter* | 0.16 | 0.00 | 0.02 | 0.04 | 0.03 | 0.01 |
| Proteobacteria | Deltaproteobacteria_unclassified | 0.04 | 0.08 | 6.69 | 1.23 | 0.00 | 0.00 |
| | ***Geobacter*** | 0.35 | 0.61 | 15.18 | 46.53 | 95.42 | 97.45 |
| | *Smithella* | 0.02 | 0.23 | 0.08 | 0.06 | 0.01 | 0.00 |
| | *Syntrophus* | 0.77 | 0.02 | 0.00 | 0.00 | 0.00 | 0.00 |
| | *Desulfitobacterium* | 0.11 | 0.07 | 0.08 | 0.09 | 0.00 | 0.00 |
| | *Desulfovibrio* | 0.30 | 0.01 | 0.00 | 0.00 | 0.00 | 0.00 |
| Spirochaetes | *Treponema* | 6.45 | 0.00 | 0.00 | 0.00 | 0.01 | 0.01 |
| | *Sphaerochaeta* | 1.96 | 0.02 | 0.01 | 0.01 | 0.05 | 0.05 |
| Tenericutes | *Acholeplasma* | 0.76 | 0.00 | 0.02 | 0.01 | 0.00 | 0.00 |
| | Bacteria_others | 1.78 | 2.78 | 3.58 | 0.71 | 0.03 | 0.03 |
| Euryarchaeota | *Methanosaeta* | 1.68 | 14.18 | 0.01 | 0.06 | 0.00 | 0.01 |
| | *Methanobacterium* | 0.56 | 0.05 | 0.12 | 0.22 | 0.00 | 0.00 |

**Fig. S2. The microbial community composition of PCE-dechlorinating microcosms in different transfers.** Only genera with relative abundances higher than 0.05% in one or more of the microcosms are shown.

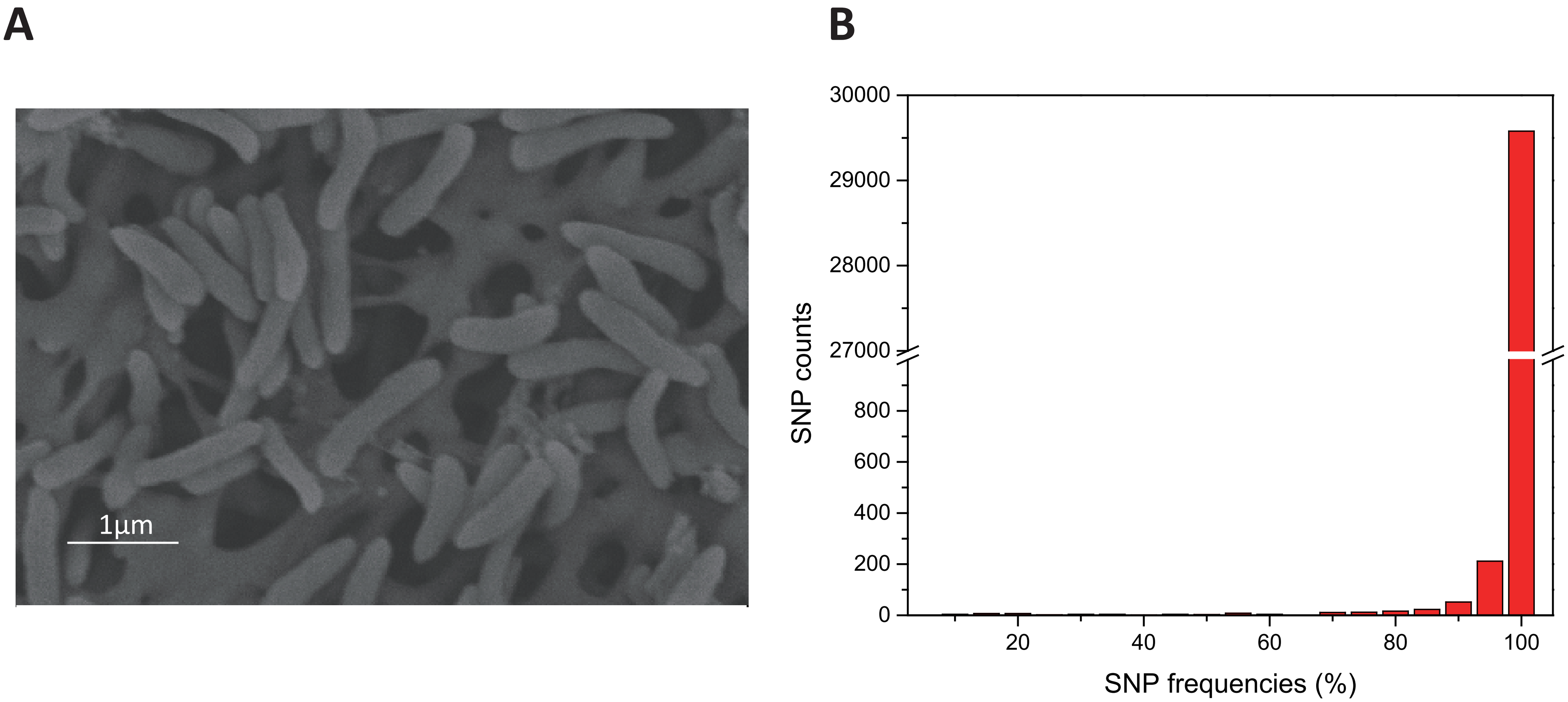


**Fig. S3. Conformation of culture purity with SEM and SNP profiling analyses. (A)** SEM image of pure culture LYY; **(B)** SNP profile of strain LYY. SNPs were called against the reference strain SZ. The sole peaks at 100% frequency of culture LYY suggest a single *Geobacter* strain.

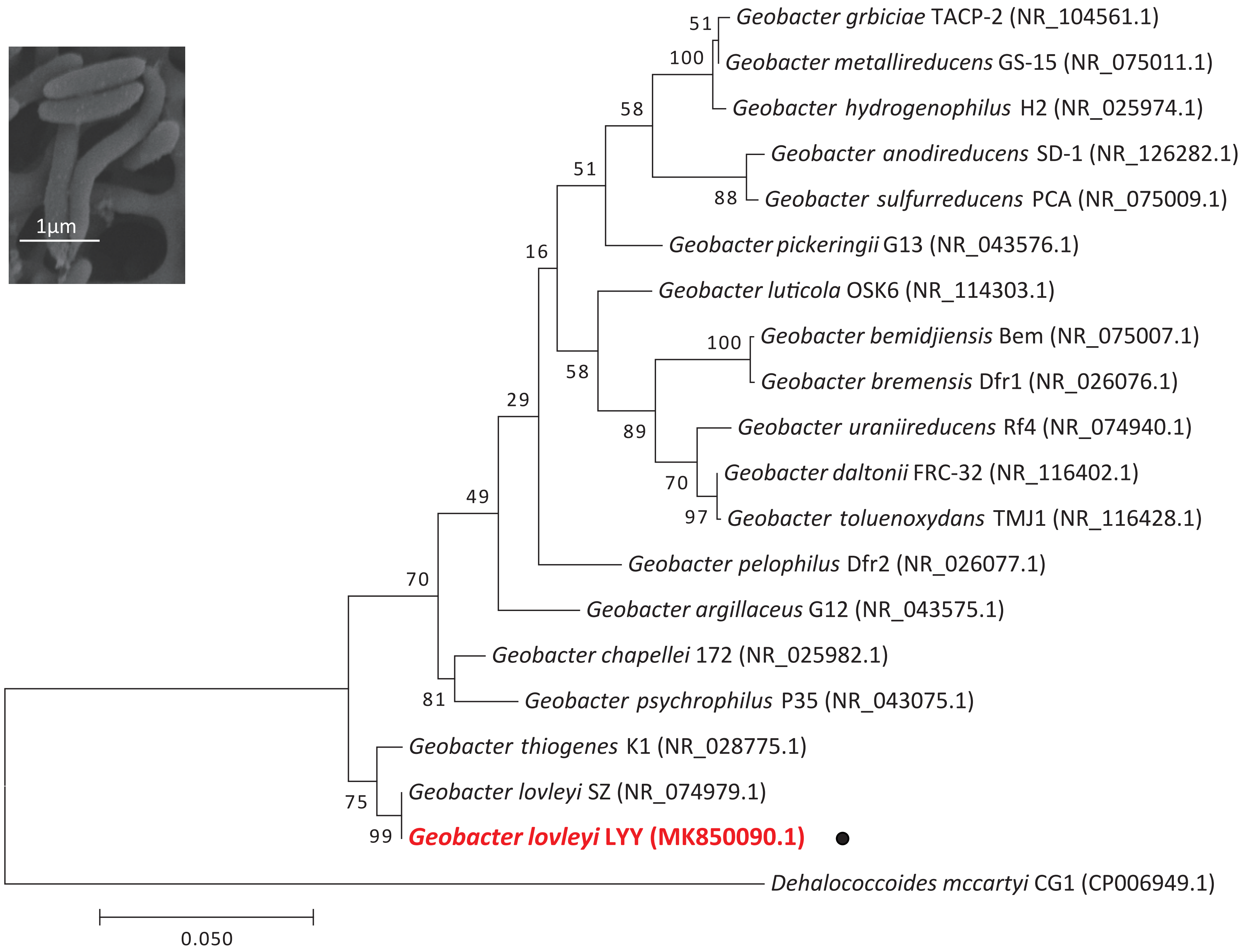


**Fig. S4. Phylogeny of *Geobacter lovleyi* LYY.** The phylogenetic tree of strain LYY and related strains has been constructed with neighbor-joining method (MEGA 7) based on their full-length 16S rRNA gene sequences. The bootstrap values at nodes are based on 1,000 replicates. The scale bar represents a 5-bp substitution per 100 nucleotides. A SEM image inside figure shows the cell morphology of *G. lovleyi* LYY.

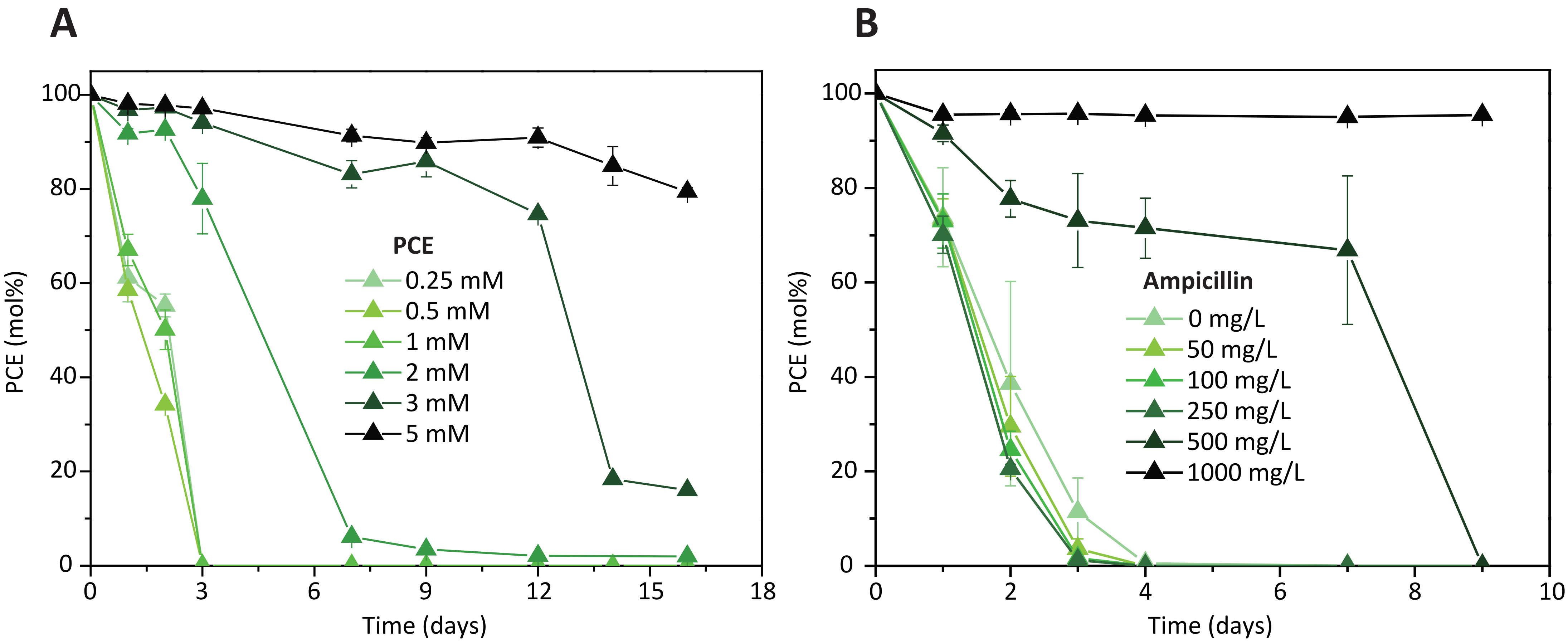


**Fig. S5 PCE dechlorination in *Geobacter lovleyi* LYY pure cultures. (A)** PCE-dechlorinating pure cultures fed with 0.25-5 mM PCE. **(B)** PCE-dechlorinating pure cultures amended with 0-1000 mg/L ampicillin and 1 mM PCE. Error bars represent SDs of triplicate cultures.

**A**

| | *G. lovleyi* | | *G. thiogenes* | *G. sulfurreducens* |
|---|---|---|---|---|
| | **LYY** | **SZ** | **K1** | **PCA** |
| Size (Mb) | 3.7 | 3.9 | 3.6 | 3.8 |
| G+C Contant (%) | 54.9 | 54.8 | 55.1 | 60.9 |
| Genes (total) | 3572 | 3726 | 3369 | 3490 |
| CDSs (total) | 3521 | 3672 | 3308 | 3432 |
| tRNA | 42 | 44 | 48 | 48 |
| rRNA (5S, 16S, 23S) | 2 | 2 | 3 | 2 |
| *rdhA* genes | 1 | 2 | N.I. | 0 |
| Plasmid (Size, bp) | 1 (81261) | 1 (77113) | 0 | 0 |

N.I.: Not identified

**B**

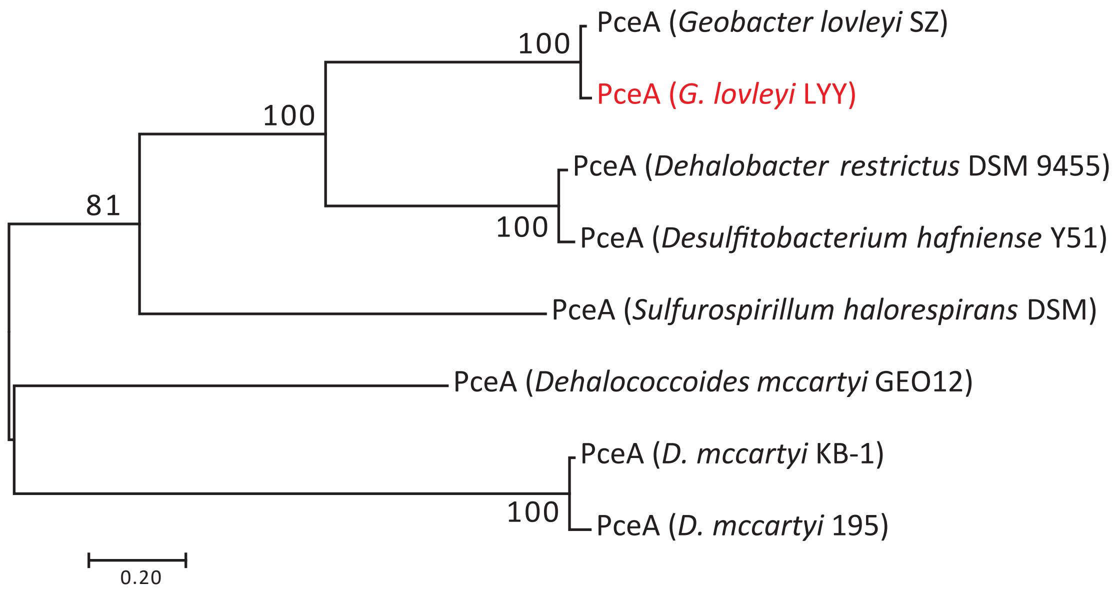


**C**

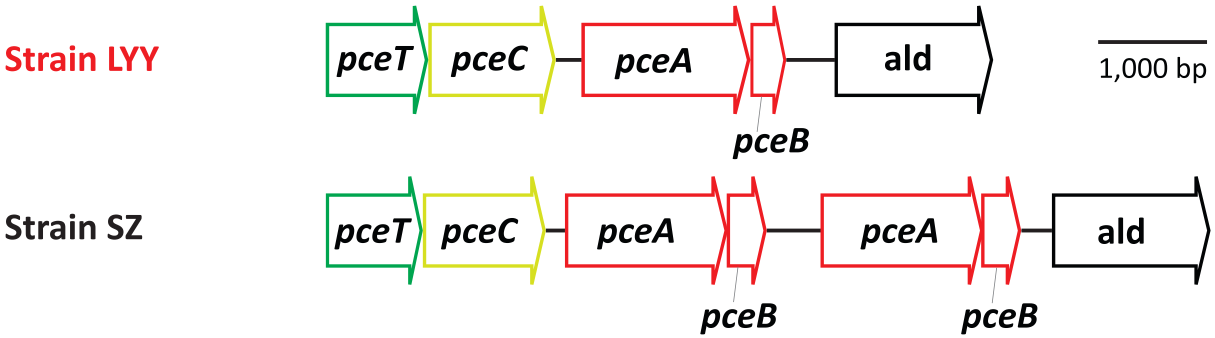


**Fig. S6. Genomic characterization of *Geobacter lovleyi* LYY. (A)** Overview of genomes of strain LYY and previously sequenced isolates (i.e., SZ, K1 and PCA) belonging to the Geobacter genus. **(B)** Phylogeny of PCE reductive dehalogenases (PceA) in OHRB of different lineages (*Geobacter lovleyi* SZ, WP_012470907.1; *Dehalobacter restrictus* DSM 9455, CAD28792.1; *Desulfitobacterium hafniense* Y51, BAC00915.1; *Sulfurospirillum halorespirans* DSM, AHJ12791.1.; *Dehalococcoides mccartyi* GEO12, POZ59159.1; *D. mccartyi* KB-1, AQX75192.1; *D. mccartyi* 195, WP_010936099.1). The tree has been generated for 550-aa aligned positions using Clustal W (MEGA 7), and bootstrap values at nodes are based on 1,000 replicates. The scale bar represents changes per amino acid position. **(C)** PCE reductive dehalogenase-encoding genomic loci on the chromosomes of *G. lovleyi* LYY and SZ.

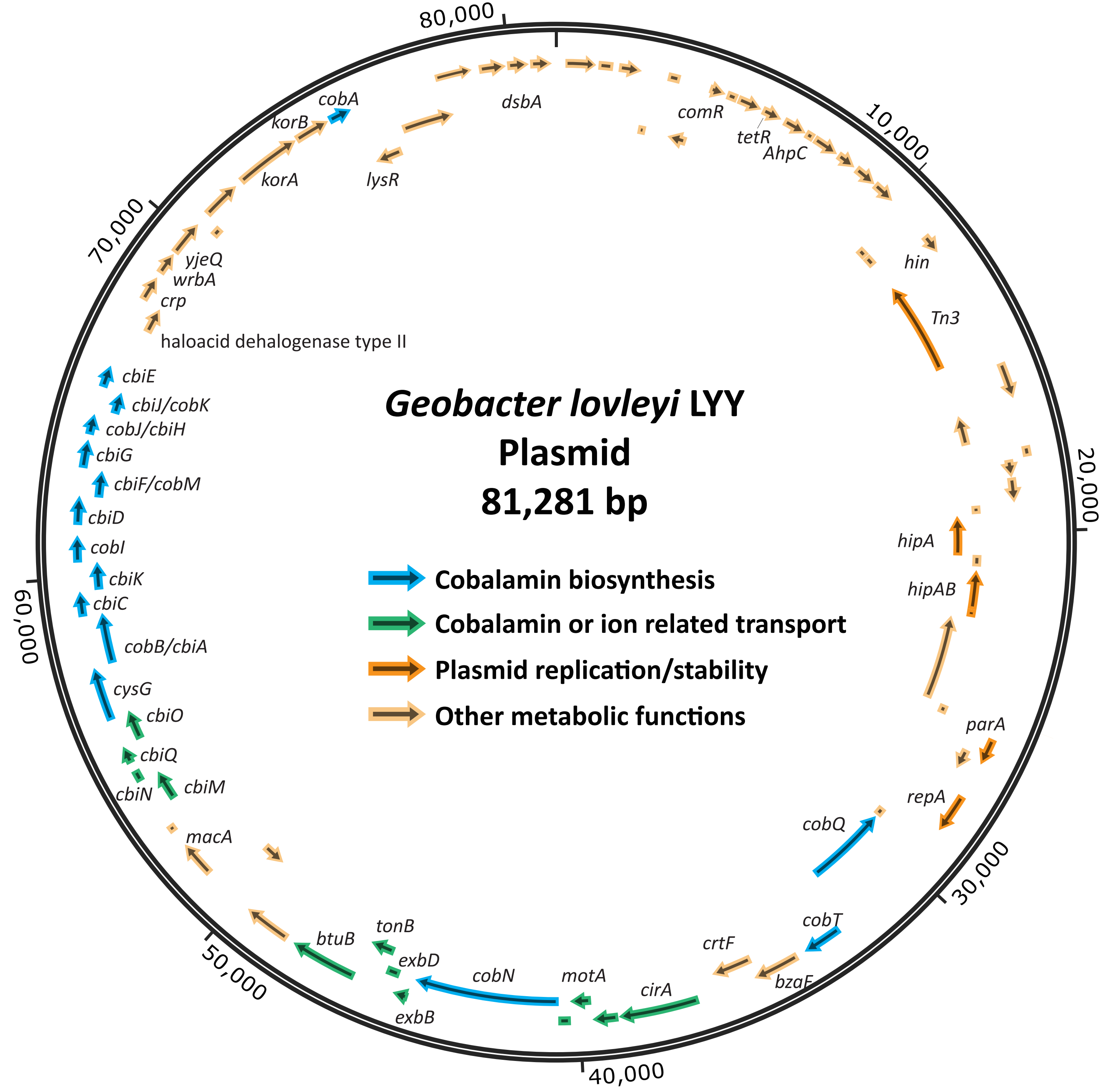


**Fig. S7. A plasmid map of *Geobacter lovleyi* LYY.** Genes for cobalamin synthesis and transport are shown in blue and green colors, respectively.

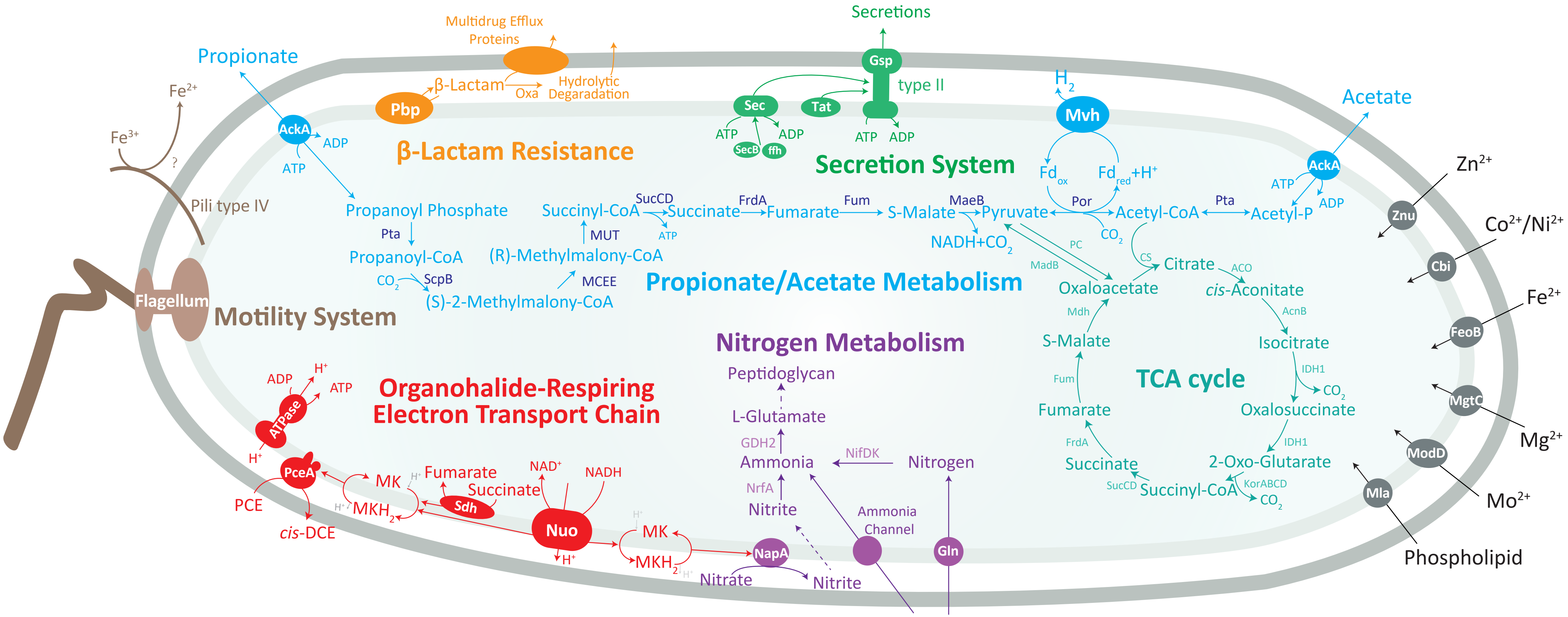


**Fig. S8. Predicted metabolic capabilities of *Geobacter lovleyi* LYY.** Metabolic pathways include organohalide-respiration (red) , propionate-to-acetate/hydrogen metabolism (blue), TCA cycle (cyan) and nitrogen metabolism (purple), as well as type II secretion systems (orange) and β-lactam resistance (blue). See **Table S3** for detailed information on genes assigned to specific metabolic pathways.

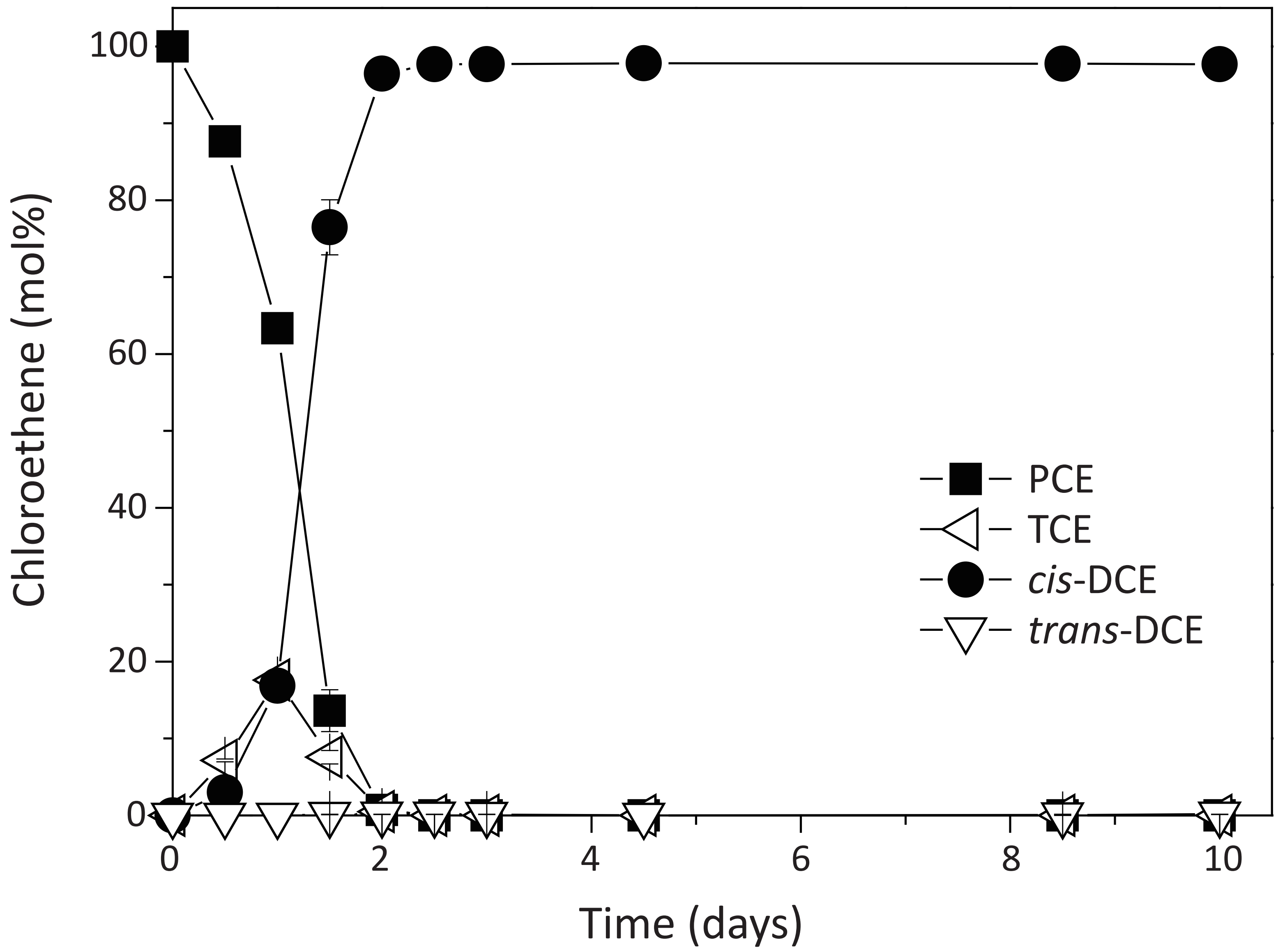


**Fig. S9. PCE dechlorination in a *Geobacter-Dehalococcoides* co-culture.** The coculture was prepared with *Geobacter lovleyi* LYY and *Dehalococcoides mccartyi* CG1.
