# Supplementary material for: Substrate-dependent competition and cooperation relationships between Geobacter and Dehalococcoides for their organohalide respiration
Source: ISME Commun. 2021 Jun 9;1:23. doi: 10.1038/s43705-021-00025-z (PMC9723705; doi:10.1038/s43705-021-00025-z)
Supplement: Supplementary file 2 — Supplementary Tables [file 43705_2021_25_MOESM2_ESM.pdf]

Supplementary Tables for

Substrate-dependent competition and cooperation relationships between *Geobacter* and *Dehalococcoides* for their organohalide respiration

Yongyi Liang[1]·Qihong Lu[1]·Zhiwei Liang[1]·Xiaokun Liu[1]·Wenwen Fang[1]·Dawei Liang[2]·Jialiang Kuang[3]·Rongliang Qiu[1,4]·Zhili He[1]·Shanquan Wang[1*]

[1] Environmental Microbiomics Research Center, School of Environmental Science and Engineering, Guangdong Provincial Key Laboratory of Environmental Pollution Control and Remediation Technology, Southern Marine Science and Engineering Guangdong Laboratory (Zhuhai), Sun Yat-Sen University, Guangzhou, China 510006

[2] Beijing Key Laboratory of Bio-inspired Energy Materials and Devices, School of Space & Environment, Beihang University, Beijing, China 100191

[3] Institute for Environmental Genomics and Department of Microbiology and Plant Biology, University of Oklahoma, Norman, OK, USA 73019

[4] Guangdong Laboratory for Lingnan Modern Agriculture, College of Natural Resources and Environment, South China Agricultural University, Guangzhou, China 510642

[*]Corresponding author: Shanquan Wang (wangshanquan@mail.sysu.edu.cn)

Table S1. Comparison of cell growth rate and yield of obligate and non-obligate PCE-dechlorinating OHRBs

OHRBs		Dechlorination products	Initial PCE concentration (mM)	Yield (cell numbers×10^8·μmol Cl^{-1})[b]		References
				Specific	Average	
Obligate	*Dehalococcoides mccartyi* 195	VC, Ethene[a]	~0.85	2.9	1.252 (0.56-2.9)	[1]
	Dehalococcoides mccartyi CBDB1	*cis*-DCE, *trans*-DCE	~0.05	1.3		[2]
	Dehalococcoides mccartyi 11a5	*cis*-DCE, VC, Ethene[a]	~0.60	0.87		[3]
	Dehalococcoides mccartyi MB	*cis*-DCE, *trans*-DCE	~0.50	1		[4]
	Dehalococcoides mccartyi CG1	*cis*-DCE, *trans*-DCE	~0.70	1.2		[5]
	Dehalococcoides mccartyi CG4	*cis*-DCE, *trans*-DCE	~0.70	0.84		[5]
	Dehalococcoides mccartyi CG5	*cis*-DCE, *trans*-DCE	~0.70	0.97		[5]
	Dehalococcoides mccartyi JNA	*cis*-DCE, *trans*-DCE	~0.70	1.63		[6]
	Dehalococcoides mccartyi CG3	*cis*-DCE, *trans*-DCE	~0.70	/		[7]
	Dehalococcoides mccartyi SG1	*cis*-DCE, *trans*-DCE	~0.70	/		[7]
	Dehalobacter restrictus PER-K23	*cis*-DCE	~10.00	~0.56		[8,9]
	Dehalobacter restrictus TEA	*cis*-DCE	~1.50	/		[10]
Non-obligate	*Desulfitobacterium hafniense* TCE1	*cis*-DCE	~15.00	0.55	0.386 (0.19-0.55)	[11]
	Desulfitobacterium hafniense Y51	*cis*-DCE	0.60	/		[12]
	Desulfitobacterium hafniense JH1	*cis*-DCE	~0.21	/		[13]
	Desulfitobacterium sp. KBC1	TCE	0.30	/		[14]
	Desulfitobacterium sp. PCE1	TCE	10.00	~0.43		[15]
	Sulfurospirillum multivorans K	*cis*-DCE	0.36	~0.47		[16]
	Geobacter lovleyi SZ	*cis*-DCE	~1.00	~0.29		[17]
	Desulfuromonas michiganensis BB1	*cis*-DCE	~0.175	~0.19		[18]

Note:
a. reductive dechlorination of VC to ethene is considered to proceed in a cometabolic process
b. cell yield, if no available data, were caluculated according to the cell density and amounts of removed chlorines.

Table S2. Substrate characterization of *Geobacter lovleyi* LYY and comparison with other *Geobacter*

**Electron donors**	**strain LYY**	**strain SZ[19]**	**strain K1[20]**	**strain 172[21]**	**strain P35[22]**	**strain G12[23]**	**strain Drf2[24]**
H_2[a]	×	√	√	×	×	×	√
Formate	×	×	×	√	√	NA	√
Acetate	√	√	√	√	√	√	√
Propionate	√	×	×	×	×	NA	√
Butyrate	×	×	×	NA	×	√	×
Lactate	×	×	×	√	NA	√	×
Citrate	×	×	NA	NA	NA	NA	NA
Pyruvate	×	√	×	NA	√	√	√
Glucose	×	×	NA	NA	NA	NA	NA
**Electron acceptors**	**strain LYY**	**strain SZ**	**strain K1**	**strain 172**	**strain P35**	**strain G12**	**strain Drf2**
PCE	√	√	NA	NA	NA	NA	NA
TCE	√	√	NA	NA	NA	NA	NA
DCE	×	×	NA	NA	NA	NA	NA
TCAc[b]	NA	NA	√	NA	NA	NA	NA
Fe(III)	√	√	√	√	√	√	√
Nitrate	√	√	√	×	×	√	×
Nitrite	×	×	NA	NA	NA	×	×
Sulfate	×	×	×	NA	×	×	×

Note:
NA, Not Available
×, no activity; √, active
a. Acetate (0.1mM) was provided as carbon source.
b. TCAc, Trichloroacetate.

Table S3. Genes assigned to specific metabolic pathways of *Geobacter lovleyi* LYY

Locus	Gene	Definition	Metabolic pathway
HHJ92_000201	*acnB*	aconitate hydratase 2 / 2-methylisocitrate dehydratase	TCA cycle
HHJ92_000525	*sdhB*	succinate dehydrogenase / fumarate reductase, iron-sulfur subunit	
HHJ92_000526	*sdhA*	succinate dehydrogenase / fumarate reductase, flavoprotein subunit	
HHJ92_000527	*sdhC*	succinate dehydrogenase / fumarate reductase, cytochrome b subunit	
HHJ92_001452	*korC*	2-oxoglutarate ferredoxin oxidoreductase subunit gamma	
HHJ92_001453	*korB*	2-oxoglutarate/2-oxoacid ferredoxin oxidoreductase subunit beta	
HHJ92_001452	*korA*	2-oxoglutarate/2-oxoacid ferredoxin oxidoreductase subunit alpha	
HHJ92_001455	*korD*	2-oxoglutarate ferredoxin oxidoreductase subunit delta	
HHJ92_001749	*fumA*	fumarate hydratase class I	
HHJ92_001851	*sucC*	succinyl-CoA synthetase beta subunit	
HHJ92_001852	*sucD*	succinyl-CoA synthetase alpha subunit	
HHJ92_002285	CS	citrate synthase	
HHJ92_002385	DLST	2-oxoglutarate dehydrogenase E2 component	
HHJ92_002386	DLD	dihydrolipoamide dehydrogenase	
HHJ92_002387	ACO	aconitate hydratase	
HHJ92_002440	*frdA*	fumarate reductase flavoprotein subunit	
HHJ92_002566	*fumC*	fumarate hydratase, class II	
HHJ92_003386	OGDH	2-oxoglutarate dehydrogenase	
HHJ92_003396	*mdh*	malate dehydrogenase	
HHJ92_003397	IDH1	isocitrate dehydrogenase	
HHJ92_000074	*ackA*	acetate kinase	Propionate /Acetate Metabolism
HHJ92_000201	*acnB*	aconitate hydratase 2 / 2-methylisocitrate dehydratase	
HHJ92_001303	*mgsA*	methylglyoxal synthase	
HHJ92_001402	*gldA*	glycerol dehydrogenase	
HHJ92_001533	*acuI*	acrylyl-CoA reductase	
HHJ92_001568	*pta*	phosphate acetyltransferase	
HHJ92_001851	*sucC*	succinyl-CoA synthetase beta subunit	
HHJ92_001852	*sucD*	succinyl-CoA synthetase alpha subunit	
HHJ92_001858	*accA*	acetyl-CoA carboxylase carboxyl transferase subunit alpha	
HHJ92_002284	*scpB*	methylmalonyl-CoA decarboxylase	
HHJ92_002372	*accB*	acetyl-CoA carboxylase biotin carboxyl carrier protein	
HHJ92_002372	*accC*	acetyl-CoA carboxylase, biotin carboxylase subunit	
HHJ92_002386	DLD	dihydrolipoamide dehydrogenase	
HHJ92_002701	*pta*	phosphate acetyltransferase	
HHJ92_002727	*accD*	acetyl-CoA carboxylase carboxyl transferase subunit beta	
HHJ92_002930	MCEE	methylmalonyl-CoA/ethylmalonyl-CoA epimerase	
HHJ92_002933	MUT	methylmalonyl-CoA mutase	
HHJ92_003112	*yqhD*	NADP-dependent alcohol dehydrogenase	
HHJ92_000194	*glnA*	glutamine synthetase	Nitrogen Metabolism
HHJ92_000315	*cynT*	carbonic anhydrase	
HHJ92_001051	*ncd2*	nitronate monooxygenase	
HHJ92_001259	*gdhA*	glutamate dehydrogenase	
HHJ92_001272	*hcp*	hydroxylamine reductase	

HHJ92_001335	*nifK*	nitrogenase molybdenum-iron protein beta chain	
HHJ92_001336	*nifD*	nitrogenase molybdenum-iron protein alpha chain	
HHJ92_001337	*nifH*	nitrogenase iron protein	
HHJ92_001381	GDH2	glutamate dehydrogenase	
HHJ92_001553	*gltD*	glutamate synthase (NADPH) small chain	
HHJ92_001554	GLU	glutamate synthase (ferredoxin)	
HHJ92_002538	*nrfA*	nitrite reductase	
HHJ92_002539	*nrfH*	cytochrome c nitrite reductase small subunit	
HHJ92_002541	*hao*	hydroxylamine dehydrogenase	
HHJ92_002553	*napA*	nitrate reductase	
HHJ92_000663	*mrdA*	penicillin-binding protein 2	β-Lactam Resistance
HHJ92_000994	*acrA*	membrane fusion protein, multidrug efflux system	
HHJ92_001363	*ftsI*	cell division protein FtsI (penicillin-binding protein 3)	
HHJ92_001967	*nagZ*	beta-N-acetylhexosaminidase	
HHJ92_002005	*mrcA*	penicillin-binding protein 1A	
HHJ92_003105	*oprM*	outer membrane protein, multidrug efflux system	
HHJ92_003106	*acrB*	multidrug efflux pump	
HHJ92_003388	*tolC*	outer membrane protein	
HHJ92_000020	*tatA*	sec-independent protein translocase protein	Secretion System
HHJ92_000326	*secF*	preprotein translocase subunit	
HHJ92_000327	*secD*	preprotein translocase subunit SecD	
HHJ92_000328	*yajC*	preprotein translocase subunit	
HHJ92_000665	*secA*	preprotein translocase subunit	
HHJ92_000707	*SPR54*	signal recognition particle subunit	
HHJ92_000788	*gspE*	general secretion pathway protein E	
HHJ92_002239	*secE*	preprotein translocase subunit SecE	
HHJ92_002272	*secY*	preprotein translocase subunit	
HHJ92_002732	*tatC*	sec-independent protein translocase protein	
HHJ92_002763	*gspM*	general secretion pathway protein M	
HHJ92_002764	*gspL*	general secretion pathway protein L	
HHJ92_002765	*gspK*	general secretion pathway protein K	
HHJ92_002766	*gspJ*	general secretion pathway protein J	
HHJ92_002767	*gspI*	general secretion pathway protein I	
HHJ92_002768	*gspH*	general secretion pathway protein H	
HHJ92_002769	*gspG*	general secretion pathway protein G	
HHJ92_002770	*gspF*	general secretion pathway protein F	
HHJ92_002772	*gspD*	type II secretion system secretin	
HHJ92_002773	*gspC*	general secretion pathway protein C	
HHJ92_003163	*secB*	preprotein translocase subunit	
HHJ92_003169	*ftsY*	fused signal recognition particle receptor	
HHJ92_003321	*yidC*	YidC/Oxa1 family membrane protein insertase	
HHJ92_003388	*tolC*	outer membrane protein	
HHJ92_003403	*secG*	preprotein translocase subunit	
HHJ92_001604	*cysK*	cysteine synthase	Sulfur Metabolism
HHJ92_001606	*metB*	cystathionine gamma-synthase	

HHJ92_001750	*cysE*	serine O-acetyltransferase	
HHJ92_001797	*ssuA*	sulfonate transport system substrate-binding protein	
HHJ92_002120	*hydB*	sulfhydrogenase subunit beta (sulfur reductase)	
HHJ92_002122	*hydG*	sulfhydrogenase subunit gamma (sulfur reductase)	
HHJ92_002225	*mddA*	methanethiol S-methyltransferase	
HHJ92_002641	*dmsB*	dimethyl sulfoxide reductase iron-sulfur subunit	
HHJ92_002654	*TST*	thiosulfate/3-mercaptopyruvate sulfurtransferase	
HHJ92_002656	*ttrB*	tetrathionate reductase subunit B	
HHJ92_002657	*phsA*	thiosulfate reductase / polysulfide reductase chain A	
HHJ92_003019	*dmsC*	dimethyl sulfoxide reductase membrane subunit	
HHJ92_003151	*metX*	homoserine O-acetyltransferase/O-succinyltransferase	
HHJ92_003350	*nrnA*	bifunctional oligoribonuclease and PAP phosphatase	
HHJ92_000503	*bchE*	anaerobic magnesium-protoporphyrin IX monomethyl ester cyclase	Cobalamin synthesis
HHJ92_000615	*ahbD*	AdoMet-dependent heme synthase	
HHJ92_000940	*bfr*	bacterioferritin	
HHJ92_001195	*cobA-hemD*	uroporphyrinogen III methyltransferase / synthase	
HHJ92_001196	*hemC*	hydroxymethylbilane synthase	
HHJ92_001197	*hemA*	glutamyl-tRNA reductase	
HHJ92_001213	PPOX	protoporphyrinogen/coproporphyrinogen III oxidase	
HHJ92_001271	EARS	glutamyl-tRNA synthetase	
HHJ92_001378	*hemB*	porphobilinogen synthase	
HHJ92_001382	cobA	cob(I)alamin adenosyltransferase	
HHJ92_001617	*cobP*	adenosylcobinamide kinase / adenosylcobinamide-phosphate guanylyltransferase	
HHJ92_001618	*cobT*	nicotinate-nucleotide--dimethylbenzimidazole phosphoribosyltransferase	
HHJ92_001619	*cobS*	adenosylcobinamide-GDP ribazoletransferase	
HHJ92_001620	*cobC*	alpha-ribazole phosphatase	
HHJ92_002178	*cobL*	precorrin-6Y C5,15-methyltransferase (decarboxylating)	
HHJ92_002179	*cobK-cbiJ*	precorrin-6A/cobalt-precorrin-6A reductase	
HHJ92_002180	*cbiG*	cobalt-precorrin 5A hydrolase	
HHJ92_002181	*cbiG*	cobalt-precorrin 5A hydrolase	
HHJ92_002182	*cobM*	precorrin-4/cobalt-precorrin-4 C11-methyltransferase	
HHJ92_002183	*cbiD*	cobalt-precorrin-5B (C1)-methyltransferase	
HHJ92_002184	*cobI-cbiL*	precorrin-2/cobalt-factor-2 C20-methyltransferase	
HHJ92_002185	*cbiK*	sirohydrochlorin cobaltochelatase	
HHJ92_002186	*cobH-cbiC*	precorrin-8X/cobalt-precorrin-8 methylmutase	
HHJ92_002187	*cobB-cbiA*	cobyrinic acid a,c-diamide synthase	
HHJ92_002188	*cysG*	uroporphyrin-III C-methyltransferase / precorrin-2 dehydrogenase / sirohydrochlorin ferrochelatase	
HHJ92_002809	*hemL*	glutamate-1-semialdehyde 2,1-aminomutase	
HHJ92_002915	*hemE*	uroporphyrinogen decarboxylase	
HHJ92_003063	*hemN*	oxygen-independent coproporphyrinogen III oxidase	
HHJ92_003225	*cbiB*	adenosylcobinamide-phosphate synthase	
HHJ92_003226	*cobQ*	adenosylcobyric acid synthase	
HHJ92_003444	*hemH*	protoporphyrin/coproporphyrin ferrochelatase	
HHJ92_003533	*cobN*	cobaltochelatase	
HHJ92_000387	*ppk*	polyphosphate kinase	

HHJ92_000525	*sdhB*	succinate dehydrogenase / fumarate reductase, iron-sulfur subunit	Organohalide-Respiring Electron Transport Chain
HHJ92_000526	*sdhA*	succinate dehydrogenase / fumarate reductase, flavoprotein subunit	
HHJ92_000527	*sdhC*	succinate dehydrogenase / fumarate reductase, cytochrome b subunit	
HHJ92_000819	*ppaC*	manganese-dependent inorganic pyrophosphatase	
HHJ92_001671	*hoxU*	bidirectional [NiFe] hydrogenase diaphorase subunit	
HHJ92_001672	*hoxF*	bidirectional [NiFe] hydrogenase diaphorase subunit	
HHJ92_001673	*hoxE*	bidirectional [NiFe] hydrogenase diaphorase subunit	
HHJ92_002440	*frdA*	fumarate reductase flavoprotein subunit	
HHJ92_002529	*ndh*	NADH dehydrogenase	
HHJ92_002687	*MQCRB*	menaquinol-cytochrome c reductase cytochrome b subunit	
HHJ92_002669	*cydA*	cytochrome bd ubiquinol oxidase subunit I	
HHJ92_002700	*cydB*	cytochrome bd ubiquinol oxidase subunit II	
HHJ92_002795	*nuoN*	NADH-quinone oxidoreductase subunit N	
HHJ92_002796	*nuoM*	NADH-quinone oxidoreductase subunit M	
HHJ92_002797	*nuoL*	NADH-quinone oxidoreductase subunit L	
HHJ92_002798	*nuoK*	NADH-quinone oxidoreductase subunit K	
HHJ92_002799	*nuoN*	NADH-quinone oxidoreductase subunit N	
HHJ92_002800	*nuoI*	NADH-quinone oxidoreductase subunit I	
HHJ92_002801	*nuoH*	NADH-quinone oxidoreductase subunit H	
HHJ92_002802	*nuoG*	NADH-quinone oxidoreductase subunit G	
HHJ92_002803	*nuoF*	NADH-quinone oxidoreductase subunit F	
HHJ92_002804	*nuoE*	NADH-quinone oxidoreductase subunit E	
HHJ92_002805	*nuoD*	NADH-quinone oxidoreductase subunit D	
HHJ92_002806	*nuoC*	NADH-quinone oxidoreductase subunit C	
HHJ92_002807	*nuoB*	NADH-quinone oxidoreductase subunit B	
HHJ92_002808	*nuoA*	NADH-quinone oxidoreductase subunit A	
HHJ92_002812	*ATPF0A*	F-type H+-transporting ATPase subunit a	
HHJ92_002813	*ATPF0C*	F-type H+-transporting ATPase subunit c	
HHJ92_002839	*ATPF1E*	F-type H+-transporting ATPase subunit epsilon	
HHJ92_002840	*ATPF1B*	F-type H+/Na+-transporting ATPase subunit beta	
HHJ92_002841	*ATPF1G*	F-type H+-transporting ATPase subunit gamma	
HHJ92_002842	*ATPF1A*	F-type H+/Na+-transporting ATPase subunit alpha	
HHJ92_002843	*ATPF1D*	F-type H+-transporting ATPase subunit delta	
HHJ92_002844	*ATPF0B*	F-type H+-transporting ATPase subunit b	
HHJ92_003483	*pceB*	membrane anchor protein subunit	
HHJ92_003484	*pceA*	reductive dehalogenase	
HHJ92_003485	*pceC*	FMN-binding protein	
HHJ92_003486	*pceT*	FKBP-type peptidyl-prolyl cis-trans isomerase	
HHJ92_000868	*motB*	chemotaxis protein	Motility system
HHJ92_000869	*motA*	chemotaxis protein	
HHJ92_001192	*flgM*	negative regulator of flagellin synthesis	
HHJ92_002947	*flgL*	flagellar hook-associated protein 3	
HHJ92_002948	*flgK*	flagellar hook-associated protein 1	
HHJ92_002951	*flgI*	flagellar P-ring protein precursor	
HHJ92_002952	*flgH*	flagellar L-ring protein precursor	

HHJ92_002953	*flgA*	flagella basal body P-ring formation protein	
HHJ92_002954	*flgG*	flagellar basal-body rod protein	
HHJ92_002956	*fliA*	RNA polymerase sigma factor for flagellar operon	
HHJ92_002959	*flhA*	flagellar biosynthesis protein	
HHJ92_002960	*flhB*	flagellar biosynthetic protein	
HHJ92_002961	*fliR*	flagellar biosynthetic protein	
HHJ92_002962	*fliQ*	flagellar biosynthetic protein	
HHJ92_002963	*fliP*	flagellar biosynthetic protein	
HHJ92_002964	*fliOZ*	flagellar protein	
HHJ92_002965	*fliNY*	flagellar motor switch protein	
HHJ92_002966	*fliM*	flagellar motor switch protein	
HHJ92_002968	*flgE*	flagellar hook protein	
HHJ92_002970	*flgD*	flagellar basal-body rod modification protein	
HHJ92_002973	*fliJ*	flagellar FliJ protein	
HHJ92_002974	*fliI*	flagellum-specific ATP synthase	
HHJ92_002975	*fliH*	flagellar assembly protein	
HHJ92_002976	*fliG*	flagellar motor switch protein	
HHJ92_002977	*fliF*	flagellar M-ring protein	
HHJ92_002978	*fliE*	flagellar hook-basal body complex protein	
HHJ92_002979	*flgC*	flagellar basal-body rod protein	
HHJ92_002980	*flgB*	flagellar basal-body rod protein	
HHJ92_003044	*fliC*	flagellin	
HHJ92_000146	*znuA*	zinc transport system substrate-binding protein	Transporters
HHJ92_000147	*znuC*	zinc transport system ATP-binding protein	
HHJ92_000148	*znuB*	zinc transport system permease protein	
HHJ92_000688	*feoB*	ferrous iron transport protein B	
HHJ92_001141	*modD*	molybdenum transport protein	
HHJ92_001899	*cbiO*	cobalt/nickel transport system ATP-binding protein	
HHJ92_001900	*cbiQ*	cobalt/nickel transport system permease protein	
HHJ92_001901	*cbiM*	cobalt/nickel transport system permease protein	
HHJ92_002224	*mgtC*	putative Mg2+ transporter-C (MgtC) family protein	
HHJ92_002851	*mlaE*	phospholipid/cholesterol/gamma-HCH transport system permease protein	
HHJ92_002852	*mlaF*	phospholipid/cholesterol/gamma-HCH transport system ATP-binding protein	
HHJ92_002853	*mlaD*	phospholipid/cholesterol/gamma-HCH transport system substrate-binding protein	
HHJ92_002855	*mlaC*	phospholipid transport system substrate-binding protein	

Table S4. 86 predicted CDS in plasmid of *Geobacter lovleyi* LYY

pLYY86 locus	Inferred/Annotated Function	Length (bp)
HHJ92_003487	IS481-like element ISGlo3 family transposase	978
HHJ92_003488	tautomerase	399
HHJ92_003489	NADPH-dependent FMN reductase	627
HHJ92_003490	MTH865-like protein	237
HHJ92_003491	DUF1516 family protein	336
HHJ92_003492	TetR family transcriptional regulator, *comR1*	573
HHJ92_003493	hypothetical protein	408
HHJ92_003494	hypothetical protein	276
HHJ92_003495	3-methyladenine DNA glycosylase	684
HHJ92_003496	TetR/AcrR family transcriptional regulator, *comR2*	567
HHJ92_003497	AhpC/TSA family protein, *bcpB*	657
HHJ92_003498	hypothetical protein	249
HHJ92_003499	SDR family oxidoreductase	735
HHJ92_003500	carboxymuconolactone decarboxylase family protein	552
HHJ92_003501	Flavodoxin family protein, *vrbA*	648
HHJ92_003502	DsbA oxidoreductase	648
HHJ92_003503	transcriptional regulator	315
HHJ92_003504	TPA addicition module protein	321
HHJ92_003505	DNA recombinase family protein, *hin*	567
HHJ92_003506	Transposase Tn3	2925
HHJ92_003507	hypothetical protein	231
HHJ92_003508	Tyrosine recombinase, *xerC1*	1014
HHJ92_003509	DNA breaking-rejoining enzymes C-terminal, *xerC2*	936
HHJ92_003510	hypothetical protein	225
HHJ92_003511	hypothetical protein	510
HHJ92_003512	hypothetical protein	705
HHJ92_003513	WGR domain-containing protein	255
HHJ92_003514	type II toxin-antitoxin system, *hipA*	1245
HHJ92_003515	helix-trun-helix domain containing protein	354
HHJ92_003516	HipA N-terminal domain protein	1212
HHJ92_003517	XRE family transcriptional regulator	240
HHJ92_003518	class I SAM-dependent DNA methyltransferase	2757
HHJ92_003519	DNA-binding protein HU 1, *hupA*	282
HHJ92_003520	Cobyrinic acid ac-diamide synthase, *parA*	651
HHJ92_003521	hypothetical protein	423
HHJ92_003522	hypothetical protein	195
HHJ92_003523	Replication initiation protein, *repA*	1113
HHJ92_003524	Integration host factor subunit alpha, *ihfA*	294
HHJ92_003525	Cobyric acid synthase, *cobQ*	2592
HHJ92_003526	Nicotinate-nucleotide—DMBA phosphoribosyltransferase, *cobT*	1053
HHJ92_003527	5-hydroxybenzimidazole synthase, *bzaF*	1311
HHJ92_003528	3-hydroxy-5-methyl-1-naphthoate 3-O-methyltransferase, *crtF*	1035
HHJ92_003529	TonB-dependent receptor, *cirA*	2214
HHJ92_003530	DUF2162 domain-containing protein	702

HHJ92_003531	flageller motor protein, *motA*	597
HHJ92_003532	DUF2149 domain-containing protein	339
HHJ92_003533	Aerobic cobaltochelatase subunit, *cobN*	4101
HHJ92_003534	Biopolymer transport protein, *exbB*	435
HHJ92_003535	iopolymer transport protein, *exbD*	384
HHJ92_003536	Protein *tonB*	735
HHJ92_003537	Vitamin B12 transporter, *btuB*	1875
HHJ92_003538	IS5-like element ISGl04 family transposase	1329
HHJ92_003539	IS3-like element ISGlo6 family transposase	750
HHJ92_003540	Macrolide export protein, *macA*	1032
HHJ92_003541	IS5-like transposase	162
HHJ92_003542	hypothetical protein	228
HHJ92_003543	Cobalt transport protein, *cbiM*	678
HHJ92_003544	Cobalt transport protein, *cbiN*	333
HHJ92_003545	Cobalt transport protein, *cbiQ*	729
HHJ92_003546	Energy-coupling factor transporter ATP-binding protein, *cbiO*	861
HHJ92_003547	Uroporphyrin-III C-methyltransferase, *cysG*	1413
HHJ92_003548	Cobyrinate a,c-diamide synthase, *cbiA*	1443
HHJ92_003549	Cobalt-precorrin-8 methylmutase, *cbiC*	687
HHJ92_003550	Sirohydrochlorin cobaltochelatase, cbiK	786
HHJ92_003551	Cobalt-precorrin-2 C(20)-methyltransferase, *cobI*	738
HHJ92_003552	Cobalt-precorrin-5B C(1)-methyltransferase, *cbiD*	1077
HHJ92_003553	Cobalt-precorrin-4 C(11)-methyltransferase, *cbiF*/*cobM*	759
HHJ92_003554	Cobalt-precorrin-5A hydrolase, *cbiG*	780
HHJ92_003555	putative cobalt-factor III C(17)-methyltransferase, *cobJ*/*cbiH*	669
HHJ92_003556	Cobalt-precorrin-6A reductase, *cbiJ*/*cobK*	786
HHJ92_003557	Cobalamin biosynthesis bifunctional protein, *cbiE*	618
HHJ92_003558	(S)-2-haloacid dehalogenase type II	687
HHJ92_003559	Crp/Fnr family transcriptional regulator	711
HHJ92_003560	NADPH-dependent FMN reductase, *wrbA*	642
HHJ92_003561	Ribosomal small subunit-dependent GTPase A	1002
HHJ92_003562	hypothetical protein	291
HHJ92_003563	5-methyltetrahydropteroyltriglutamate-homocysteine methyltransferase	1002
HHJ92_003564	2-oxoglutarate oxidoreductase subunit, *korA*	1854
HHJ92_003565	2-oxoglutarate oxidoreductase subunit, *korB*	1002
HHJ92_003566	Cob(I)yrinic acid a,c-diamide adenosyltransferase, *btuR*	663
HHJ92_003567	Hca operon transcriptional activator, *lysR*	867
HHJ92_003568	P-loop_NTPase transposase	1572
HHJ92_003569	IS5376 putative ATP-binding protein	762
HHJ92_003570	Diacetyl reductase [(S)-acetoin forming] SDR family oxidoreductase, *budC*	735
HHJ92_003571	2-hydroxychromene-2-carboxylate isomerase, *dsbA*	588
HHJ92_003572	hypothetical protein	588

Table S5. Gibbs free energy of propionate and acetate transformation

**Reaction**	**ΔG$^{0'}$ (kJ/mol)**	**Reference**
Propionate$^-$ +2H_2O → Acetate$^-$ + CO_2 + 3H_2	+76.0	[25]
Propionate$^-$+ 2H_2O + 2CO_2 → Acetate$^-$+ 3$HCOO^-$+ 3H^+	+65.3	[25]
Propionate$^-$+ 7H_2O → 3HCO_3^- + 7H_2 + 2H^+	+181.1	[25]
PCE + H_2 → TCE + H^+ + Cl^-	-173.4	[26]
PCB180 + H_2 → PCB153 + H^+ + Cl^-	-115.3	[27]

Table S6. Environmental distribution of *Dehalococcoides and Geobacter*

Type of samples	Samples Description	Relative Abundance (%)[a]		References
		Dehalococcoides or Dehalococcoidaceas	*Geobacter or Geobcteraceae*	
**Core soil samples**	The open burning site of e-waste recycling village	0.08	0.68	[28]
**Core soil samples**	The adjacent farmland site of e-waste recycling village	0.08	2.19	[28]
**Core sediment samples**	The stream site near e-waste recycling village	0.43	1.54	[28]
**Soil and groundwater**	A local industrial area, with chlorinated ethenes contaminated	0.00	5.00	[29]
**Soil samples**	Remedial investigation well of high TCE concentration site	0.00	~1.02	[30]
**Biowall samples**	Biowall with low/moderate TCE contamination	~0.04	~1.30	[30]
**Biowall samples**	Biowall with high TCE contamination	~0.04	~2.26	[30]
**Biowall samples**	Biowall with moderate TCE contamination	~0.05	~1.64	[30]
**Groundwater samples**	An industrial park site with PCE/TCE contaminated	0.00	6.37	[31]
**Sediment samples**	Emergency wastewater overflow lagoon with PCB-contaminated	0.02-0.36	0.01-3.8	[32]
**Sediment samples**	Houston Ship Channel	1.00	2.00	[33]
**Sediment sample**	Wastewater lagoon contaminated with PCBs A1	0.10	0.30	[34]
**Sediment sample**	A2	0.05	3.80	[34]
**Sediment sample**	A3	0.07	1.10	[34]
**Sediment sample**	A4	0.20	1.00	[34]
**Sediment sample**	A5	0.08	1.30	[34]
**Sediment sample**	B1	0.12	2.00	[34]
**Sediment sample**	B2	0.08	0.40	[34]
**Sediment sample**	B3	0.02	0.25	[34]
**Sediment sample**	B4	0.11	0.70	[34]
**Sediment sample**	B5	0.07	0.10	[34]
**Sediment sample**	C1	0.08	0.15	[34]
**Sediment sample**	C2	0.13	1.20	[34]
**Sediment sample**	C3	0.03	0.20	[34]
**Sediment sample**	C4	0.02	0.05	[34]
**Sediment sample**	C5	0.17	1.10	[34]
**Sediment sample**	D1	0.09	0.20	[34]
**Sediment sample**	D2	0.10	0.10	[34]
**Sediment sample**	D3	0.07	0.40	[34]
**Sediment sample**	D4	0.18	0.50	[34]
**Sediment sample**	D5	0.02	0.08	[34]
**Sediment sample**	E1	0.03	0.05	[34]
**Sediment sample**	E2	0.05	0.30	[34]
**Sediment sample**	E3	0.03	0.10	[34]
**Sediment sample**	E4	0.07	0.40	[34]
**Sediment sample**	E5	0.35	1.10	[34]
**Sediment sample**	F3	0.03	1.20	[34]

**Sediment sample**	F4	0.02	3.70	[34]
**Sediment sample**	River sediment	0.05	1.80	[35]
**Groundwater sample**	PCE contaminated groundwater P2-C3	0.22	0.56	[36]
**Groundwater sample**	P2-C4	0.40	1.14	[36]
**Groundwater sample**	P2-C5	0.07	0.10	[36]
**Groundwater sample**	P3-C3	1.48	2.55	[36]
**Groundwater sample**	P3-C4	1.82	3.91	[36]
**Groundwater sample**	TCE contaminated groundwater S1	0.01	0.80	[37]
**Groundwater sample**	groundwater N5	0.01	0.16	[37]
**Sediment sample**	PCB contaminated sediment M1DNA	0.08	0.34	[38]
**Sediment sample**	sediment L1	0.55	3.55	[39]
**Sediment sample**	sediment L2	0.18	3.85	[39]
**Sediment sample**	sediment L3	2.22	0.07	[39]
**Groundwater sample**	Chlorinated solvent contaminated groundwater MW35	0.50	4.40	[40]
**Groundwater sample**	MW113	0.30	3.00	[40]
**Groundwater sample**	MW514	0.30	1.80	[40]
**Groundwater sample**	MW2	1.00	7.50	[40]
**Groundwater sample**	MW3	0.80	10.00	[40]
**Groundwater sample**	MW4	0.40	6.20	[40]
**Groundwater sample**	IW3	2.00	14.00	[40]
**Groundwater sample**	IW4	1.80	17.50	[40]
**Groundwater sample**	IW6	3.80	8.00	[40]
**Groundwater sample**	MW114	0.12	1.00	[40]
**Groundwater sample**	MW303S	0.20	1.80	[40]
**Groundwater sample**	IW5	0.50	2.30	[40]
**Groundwater sample**	IW7	0.52	1.80	[40]
**Groundwater sample**	MW38	0.50	2.00	[40]
**Groundwater sample**	MW40	0.65	3.00	[40]
**Soil sample**	Chlorinated ethenes contaminated soil MV-1	0.10	0.00	[41]
**Soil sample**	MV-2	1.94	0.00	[41]
**Soil sample**	MV-3	1.02	0.00	[41]
**Groundwater sample**	Chloroethene contaminated groundwater	0.10	1.70	[42]

Note: a, the data was calculated based on the 16S rRNA sequencing analysis

References:

[1] Maymo-Gatell X, Chien XY, Gossett JM, Zinder SH. 1997. Isolation of a bacterium that reductively dechlorinates tetrachloroethene to ethene. Science. 276:1568–1571.

[2] Marco-Urrea E, Nijenhuis I, Adrian L. 2011. Transformation and carbon isotope fractionation of tetra- and trichloroethene to trans-dichloroethene by Dehalococcoides sp. strain CBDB1. Environ Sci Technol. 45:1555–1562.

[3] Zhao, S, Ding, C, He, J, 2016. Genomic characterization of Dehalococcoides mccartyi strain 11a5 reveals a circular extrachromosomal genetic element and a new tetrachloroethene reductive dehalogenase, FEMS Microbiol Ecol 94, fiw235.

[4] Cheng D, He JZ. 2009. Isolation and characterization of "Dehalococcoides" sp. strain MB, which dechlorinates tetrachloroethene to trans-1,2-dichloroethene. Appl Environ Microbiol. 75:5910–5918.

[5] Wang S, Chng KR, Wilm A, Zhao S, Yang KL, Nagarajan N, He J. 2014. Genomic characterization of three unique Dehalococcoides that respire on persistent polychlorinated biphenyls. Proc Natl Acad Sci USA. 111:12103–12108.

[6] Wang S, Chng K R, Chen C, Bedard D L, He J. 2015. Genomic characterization of Dehalococcoides mccartyi strain JNA that reductively dechlorinates tetrachloroethene and polychlorinated biphenyls. Environ. Sci. Technol. 49:14319-14325.

[7] Wang S, Chen C, Siyan Z, Jianzhong H. 2019. Microbial synergistic interactions for reductive dechlorination of polychlorinated biphenyls. Science of Total Environment. 666: 368-376.

[8] Holliger C, Schraa G, Stams AJM, Zehnder AJB. 1993.A highly purified enrichment culture couples the reductive dechlorination of tetrachloroethene to growth. Appl Environ Microbiol. 59:2991–2997.

[9] Holliger C, Hahn D, Harmsen H, Ludwig W, Schumacher W, Tindall B, Vazquez F, Weiss N, Zehnder A J.1998. Dehalobacter restrictus gen. nov. and sp. nov., a strictly anaerobic bacterium that reductively dechlorinates tetra- and trichloroethene in an anaerobic respiration. Arch Microbiol. 169:313-321.

[10] Wild A, Hermann R, Leisinger T. 1996. Isolation of an anaerobic bacterium which reductively dechlorinates tetrachloroethene and trichloroethene. Biodegradation. 7:507–511.

[11] Gerritse J, Drzyzga O, Kloetstra G, Keijmel M, Wiersum LP, Hutson R, Collins MD, Gottschal JC. 1999. Influence of different electron donors and acceptors on dehalorespiration of tetrachloroethene by Desulfitobacterium frappieri TCE1. Appl Environ Microbiol. 65:5212–5221.

[12] Suyama A, Iwakiri R, Kai K, Tokunaga T, Sera N, Furukawa K. 2001. Isolation and characterization of Desulfitobacterium sp strain Y51 capable of efficient dehalogenation of tetrachloroethene and polychloroethanes. Biosci Biotechnol Biochem. 65:1474–1481.

[13] Fletcher KE, Ritalahti KM, Pennell KD, Takamizawa K, Löffler FE. 2008. Resolution of culture Clostridium bifermentans DPH-1 into two populations, a Clostridium sp. and tetrachloroethene-dechlorinating Desulfitobacterium hafniense strain JH1. Appl Environ Microbiol 74:6141–6143.

[14] Tsukagoshi N, Ezaki S, Uenaka T.2006. Isolation and transcriptional analysis of novel tetrachloroethene reductive dehalogenase gene from Desulfitobacterium sp. strain KBC1. Appl Microbiol Biotechnol. 69:543–553.

[15] Gerritse J, Renard V, Pedro Gomes TM, Lawson PA, Collins MD, Gottschal JC. 1996. Desulfitobacterium sp. strain PCE1, an anaerobic bacterium that can grow by reductive dechlorination of tetrachloroethene or ortho-chlorinated phenols. Arch Microbiol. 165:132–140.

[16] Scholz-Muramatsu H, Neumann A, Messmer M, Moore E, Diekert G. 1995. Isolation and characterization of Dehalospirillum multivorans gen. nov., sp. nov., a tetrachloroethene-utilizing, strictly anaerobic bacterium. Arch Microbiol. 163:48–56.

[17] Sung Y, Fletcher K, Ritalahti KM, Apkarian RP, Ramos-Hernández N, Sanford RA.2006. Geobacter lovleyi sp. nov. strain SZ, a novel metal-reducing and tetrachloroethene-dechlorinating bacterium. Appl Environ Microbiol. 72:2775–2782.

[18] Sung Y, Ritalahti KM, Sanford RA, Urbance JW, Flynn SJ, Tiedje JM, Löffler FE. 2003. Characterization of two tetrachloroethene-reducing, acetate-oxidizing anaerobic bacteria and their description as Desulfuromonas michiganensis sp. nov. Appl Environ Microbiol. 69:2964–2974.

[19] Sung Y, Fletcher K, Ritalahti KM, Apkarian RP, Ramos-Hernández N, Sanford RA.2006. Geobacter lovleyi sp. nov. strain SZ, a novel metal-reducing and tetrachloroethene-dechlorinating bacterium. Appl Environ Microbiol. 72:2775–2782.

[20] Nevin KP, Holmes DE, Woodard TL, Covalla SF, Lovley DR. 2007. Reclassification of Trichlorobacter thiogenes as Geobacter thiogenes comb. nov. Int J Syst Evol Microbiol 57:463-6.

[21] Coates JD, Bhupathiraju VK, Achenbach LA, McInerney MJ, Lovley DR. 2001. Geobacter hydrogenophilus, Geobacter chapellei and Geobacter grbiciae, three new, strictly anaerobic, dissimilatory Fe(III)-reducers. Int J Syst Evol Microbiol, 51: 581-588.

[22] Nevin KP, Holmes DE, Woodard TL, Hinlein ES, Ostendorf DW, Lovley DR. 2005. Geobacter bemidjiensis sp. nov. and Geobacter psychrophilus sp. nov., two novel Fe(III)-reducing subsurface isolates. Int J Syst Evol Microbiol, 55:1667-74.

[23] Shelobolina ES, Nevin KP, Blakeney-Hayward JD, Johnsen CV, Plaia TW, Krader P, Woodard T, Holmes DE, Vanpraagh CG, Lovley D R. 2007. Geobacter pickeringii sp. nov., Geobacter argillaceus sp. nov. and Pelosinus fermentans gen. nov., sp. nov., isolated from subsurface kaolin lenses. Int J Syst Evol Microbiol, 57: 126-35.

[24] Straub KL, Buchholz-Cleven BEE. 2001 Geobacter bremensis sp. nov. and Geobacter pelophilus sp. nov., two dissimilatory ferric-iron-reducing bacteria. Int J Syst Evol Microbiol, 51: 1805–1808.

[25] Thauer RK, Jungermann K, Decker K. 1977. Energy conservation in chemotrophic anaerobic bacteria. Bacteriol Rev 41: 100-180.

[26] Dolfing J. 2000. Energetics of anaerobic degradation pathways of chlorinated aliphatic compounds. Microb Ecol 40: 2-7.

[27] Holmes DA, Harrison BK, Dolfing J. 1993. Estimation of Gibbs free energies of formation of polychlorinated biphenyls. Environ Sci Technol 27: 725-731.

[28] Wu Q, Du Y, Huang Z, Gu J, Leung JYS, Mai B, Xiao T, Liu W, Fu J. 2019. Vertical profile of soil/sediment pollution and microbial community change by e-waste recycling operation. Sci Total Environ, 669: 1001-1010.

[29] Ana, GB, Choi J, Ahn Y. 2019. Reductive dechlorination of perchloroethene (PCE) and bacterial community changes in a continuous-flow, two-stage anaerobic column. Int Biodeter Biode 138: 41-49.

[30] Nino de Guzman GT, Hapeman CJ, Millner PD, Torrents A, Jackson D, Kjellerup BV. 2018. Presence of organohalide-respiring bacteria in and around a permeable reactive barrier at a trichloroethylene-contaminated Superfund site. Environ Pollut 243: 766-776.

[31] Kao CM, Liao HY, Chien CC, Tseng YK, Tang P, Lin CE, Chen SC. 2016. The change of microbial community from chlorinated solvent-contaminated groundwater after biostimulation using the metagenome analysis. J Hazard Mater 302:144-150.

[32] Mattes TE, Ewald JM, Liang Y, Martinez A, Awad A, Richards P, Hornbuckle KC, Schnoor JL. 2018. PCB dechlorination hotspots and reductive dehalogenase genes in sediments from a contaminated wastewater lagoon. Environ Sci Pollut Res, 25: 16376-16388.

[33] Hieke AC, Brinkmeyer R, Yeager KM, Schindler K, Zhang S, Xu C, Louchouarn P, Santschi PH. 2016. Widespread Distribution of Dehalococcoides mccartyi in the Houston Ship Channel and Galveston Bay, Texas, Sediments and the Potential for Reductive Dechlorination of PCDD/F in an Estuarine Environment. Mar Biotechnol, 18: 630-644.

[34] Mattes, T.E., Ewald, J.M., Liang, Y., Martinez, A., Awad, A., Richards, P., Hornbuckle, K.C., Schnoor, J. L. 2018. PCB dechlorination hotspots and reductive dehalogenase genes in sediments from a contaminated wastewater lagoon, Environ. Sci. Pollut. Res. 25, 16376-16388.

[35] Lee, J., Lee, T.K. 2016. Development and characterization of PCE-to-Ethene dechlorinating microcosms with contaminated river sediment. J. Microbiol. Biotechnol. 26, 120-129.

[36] Dugat-Bony, E., Biderre-Petit, C., Jaziri, F., David, M.M., Denonfoux, J., Lyon, D.Y., Richard, J., Curvers, C., Boucher, D., Vogel, T.M., Peyretaillade, E., Peyret, P. 2012. In situ TCE degradation mediated by complex dehalorespiring communities during biostimulation processes, Microbiol. Biotechnol. 5, 642-653.

[37] Sutton, N.B., Atashgahi, S., van der Wal, J., Wijin, G., Grotenhuis, T., Smidt, H., Rijinaarts, 2015. Microbial Dynamics During and After In Situ Chemical Oxidation of Chlorinated Solvents.

[38] Praveckova, M., Brennerova, M., de Alencastro, F., Holliger, C., Rossi, P. 2016. Indirect evidence link PCB dehalogenation with Geobacteraceae in anaerobic sediment-free microcosms. Front. Microbiol. 7, 933

[39] Simsir, B., Yan, J., Im, J., Graves, D., Loffler, F.E. 2017. Natural Attenuation in Streambed Sediment Receiving Chlorinated Solvents from Underlying Fracture Networks. Environ. Sci. Technol. 51, 4821-4830.

[40] Dang, H., Kanitkar, Y.H., Stedtfeld, R.D., Hatzinger, P.B., Hashsham, S.A., Cupples, A.M. 2018. Abundance of Chlorinated Solvent and 1,4-Dioxane Degrading Microorganisms at Five Chlorinated Solvent Contaminated Sites Determined via Shotgun Sequencing. Environ. Sci. Technol. 52, 13914-13924.

[41] Nemecek, J., Dolinova, I., Machackova, J., Spanek, R., Sevcu, A., Lederer, T., Cernik, M. 2017. Stratification of chlorinated ethenes natural attenuation in an alluvial aquifer assessed by hydrochemical and biomolecular tools. Chemosphere 184, 157-1167.

[42] Miura, T., Yamazoe, A., Ito, M., Ohji, S., Hosoyama, A., Takahata, Y., Fujita, N. 2015. The Impact of Injections of Different Nutrients on the Bacterial Community and Its Dechlorination Activity in Chloroethene-Contaminated Groundwater Microbes. Environ. 30, 164-171.
